# Spatiotemporal dynamics of HSV genome nuclear entry and compaction state transitions using bioorthogonal chemistry and super-resolution microscopy

**DOI:** 10.1371/journal.ppat.1006721

**Published:** 2017-11-09

**Authors:** Eiki Sekine, Nora Schmidt, David Gaboriau, Peter O’Hare

**Affiliations:** 1 Section of Virology, Department of Medicine, Imperial College, St Mary’s Medical School, London, United Kingdom; 2 Department of Medicine, Facility for Imaging by Light Microscopy, National Heart and Lung Institute, Imperial College, London, United Kingdom; University of Wisconsin-Madison, UNITED STATES

## Abstract

We investigated the spatiotemporal dynamics of HSV genome transport during the initiation of infection using viruses containing bioorthogonal traceable precursors incorporated into their genomes (HSV^EdC^). In vitro assays revealed a structural alteration in the capsid induced upon HSV^EdC^ binding to solid supports that allowed coupling to external capture agents and demonstrated that the vast majority of individual virions contained bioorthogonally-tagged genomes. Using HSV^EdC^ in vivo we reveal novel aspects of the kinetics, localisation, mechanistic entry requirements and morphological transitions of infecting genomes. Uncoating and nuclear import was observed within 30 min, with genomes in a defined compaction state (ca. 3-fold volume increase from capsids). Free cytosolic uncoated genomes were infrequent (7–10% of the total uncoated genomes), likely a consequence of subpopulations of cells receiving high particle numbers. Uncoated nuclear genomes underwent temporal transitions in condensation state and while ICP4 efficiently associated with condensed foci of initial infecting genomes, this relationship switched away from residual longer lived condensed foci to increasingly decondensed genomes as infection progressed. Inhibition of transcription had no effect on nuclear entry but in the absence of transcription, genomes persisted as tightly condensed foci. Ongoing transcription, in the absence of protein synthesis, revealed a distinct spatial clustering of genomes, which we have termed genome congregation, not seen with non-transcribing genomes. Genomes expanded to more decondensed forms in the absence of DNA replication indicating additional transitional steps. During full progression of infection, genomes decondensed further, with a diffuse low intensity signal dissipated within replication compartments, but frequently with tight foci remaining peripherally, representing unreplicated genomes or condensed parental strands of replicated DNA. Uncoating and nuclear entry was independent of proteasome function and resistant to inhibitors of nuclear export. Together with additional data our results reveal new insight into the spatiotemporal dynamics of HSV genome uncoating, transport and organisation.

## Author summary

Virtually all DNA virus classes as well as many RNA viruses must deposit their genomes within the nucleus for transcription, genome replication and subsequent capsid assembly. While infecting capsids have been studied by various methods and biochemical approaches have been used to investigate the bulk genome population characteristics, quantitative spatiotemporal information of the infecting genome itself at the single particle level has been lacking. This is required for any complete understanding of many critical aspects of virus infection and virus pathogenesis. Using novel techniques in bioorthogonal chemistry to produce normal non-recombinant viruses with readily traceable genomes, we provide the first direct quantitative spatiotemporal analysis of HSV genome transport and presentation to the cellular environment. Using these techniques which discriminate encapsidated from uncoated genomes and input from replicated DNA, our work provides a comprehensive analysis, using direct measures for genome detection not dependant on surrogate outputs. The results reveal completely novel aspects of early genome localisation and organisation not previously appreciated or amenable to study. Furthermore the work also provides a roadmap for similar studies in other systems and for future analysis of many aspects in different fields of the biology of infecting virus genomes early during cell infection.

## Introduction

Virtually all DNA virus classes including herpesviruses, adenoviruses, hepatitis B virus, parvoviruses and polyomaviruses must deposit their genomes within the nucleus for transcription, genome replication and subsequent capsid assembly. Genome transport and entry to the nucleus is also a prerequisite for replication of retroviruses, lentiviruses including HIV and certain RNA viruses including e.g., orthomyxoviruses such as influenza virus. All these viruses must navigate through the cytoplasm of infected cells, escape or counteract physical host cell barriers and antiviral processes, and engage with the nuclear envelope or nuclear pore for genome import into the nucleus [1–9]. Despite advances in certain areas [10–16] much remains to be understood concerning the detailed pathways and mechanisms involved, particularly with regard to the localisation of infecting virus genomes themselves and their regulated (or premature) presentation to the cell environment. Quantitative spatiotemporal information at the single particle level on localisation, uncoating and transport of the infecting genome is required for any complete understanding of many critical aspects of virus infection and virus pathogenesis.

Among the factors which have limited the quantitative spatiotemporal analysis of genome transport and presentation are the insensitivity or ready tractability of methods to directly visualise and measure virus genomes, the inability to differentiate genomes that are encapsidated from those that have dissociated, the inability to readily differentiate incoming from replicated genomes; and the incompatibility of certain detection methods with immunohistochemistry for parallel detection of host and viral protein components. One of the most frequently used techniques, fluorescence in situ hybridisation (FISH), has provided many advances, and yet still presents several hurdles and limitations [16]. FISH inherently cannot discriminate input from replicated genomes nor, due to the harsh conditions frequently incompatible with immunofluorescence, does FISH discriminate between encapsidated genomes versus released genomes. Other routes such as the incorporation of multimerised binding sites for fluorescent DNA binding proteins, e.g. YFP-TetR [17] offer possibilities for live cell imaging but require specialised recombinant viruses or cell lines and can still be highly limited in detecting infecting genomes. Such limitations and issues of sensitivity or tractability have meant that many studies on aspects of virus infection have almost invariably relied on indirect, surrogate measures of detection of virus genomes. Reports, e.g., on the effect of inhibitors on DNA entry or analysis of DNA sensing have inferred effects on genome localisation from protein localisation. Clearly genomes not bound by surrogate markers will not be detected for any number of reasons including unknown but specific differences between genomes, occlusion by other factors, spatial segregation dictating differential protein association, repression by chromatin, non-nuclear localisations where surrogate markers may not co-localise and many others factors. Such assays also give little information on other aspects of uncoating and the morphological state of infecting genomes. These and other considerations limit our understanding of genome entry, uncoating, nuclear translocation and physical transitions, all of which are necessary for a true understanding of the earliest processes governing virus infection and host responses.

In this regard, the development of bioorthogonal metabolic precursors combined with cycloaddition to corresponding capture reagents is increasingly being exploited in various approaches to biological processes and to mechanisms in infection and immunity [16, 18–21]. Analysis of DNA synthesis by labelling with alkyne-derivatised nucleosides and cycloaddition to azide-coupled fluorochromes has been evaluated in several systems [20, 22, 23] and used in spatial, biochemical, and systems approaches to investigate DNA replication and the cell cycle. These techniques have recently been exploited by the Greber laboratory for analysis of adenovirus (Adv) infection [16] using viruses incorporating the alkyne-derivatised nucleosides EdC (ethynyl-deoxycytidine) or EdU (ethynyl-deoxyuridine) in their genomes for the spatiotemporal investigation of genome trafficking at the single particle level. We also recently showed that EdC was efficiently incorporated into HSV replication compartments and that incubation with EdC had no significant effect on HSV plaque forming ability or spread, reflected in plaque size [24]. De novo HSV DNA synthesis has also been analysed using EdC incorporation [25].

Here we expand on these methods to produce infectious HSV containing EdC incorporating genomes, (termed HSV^EdC^). Using an in vitro uncoating assay on solid supports [26, 27], we show that the vast majority of particles contained bioorthogonally-tagged genomes, detectable by cycloaddition to azide-linked fluorescent probes. Remarkably, if HSV^EdC^ virions were also heat treated on the support prior to cycloaddition, virus DNA ejected from the capsid could be coupled to azide-linked fluorochromes and detectable as filamentous strands. Genomes were not detectable in virions in solution nor on cell surfaces at +4°C, where numerous capsids could readily be observed but without genome accessibility. When infection was initiated by raising the temperature to 37°C, DNA uncoating and transport in the nucleus could be observed within 30 mins and prior to synthesis and recruitment of the major immediate-early (IE) regulator ICP4. Using these assays we undertake a comprehensive quantitative spatiotemporal analysis of genome trafficking and uncoating, including analysis by three-dimensional structured illumination microscopy. Together with additional data reported here, this work provides the first direct quantitative spatiotemporal analysis of HSV genome transport and presentation to the cellular environment, revealing new processes in genome dynamics not previously appreciated and advancing our understanding of these crucial early steps in infection.

## Results

### DNA synthesis in uninfected and HSV infected cells analysed by ethynyl-nucleoside incorporation

**Fig 1:**
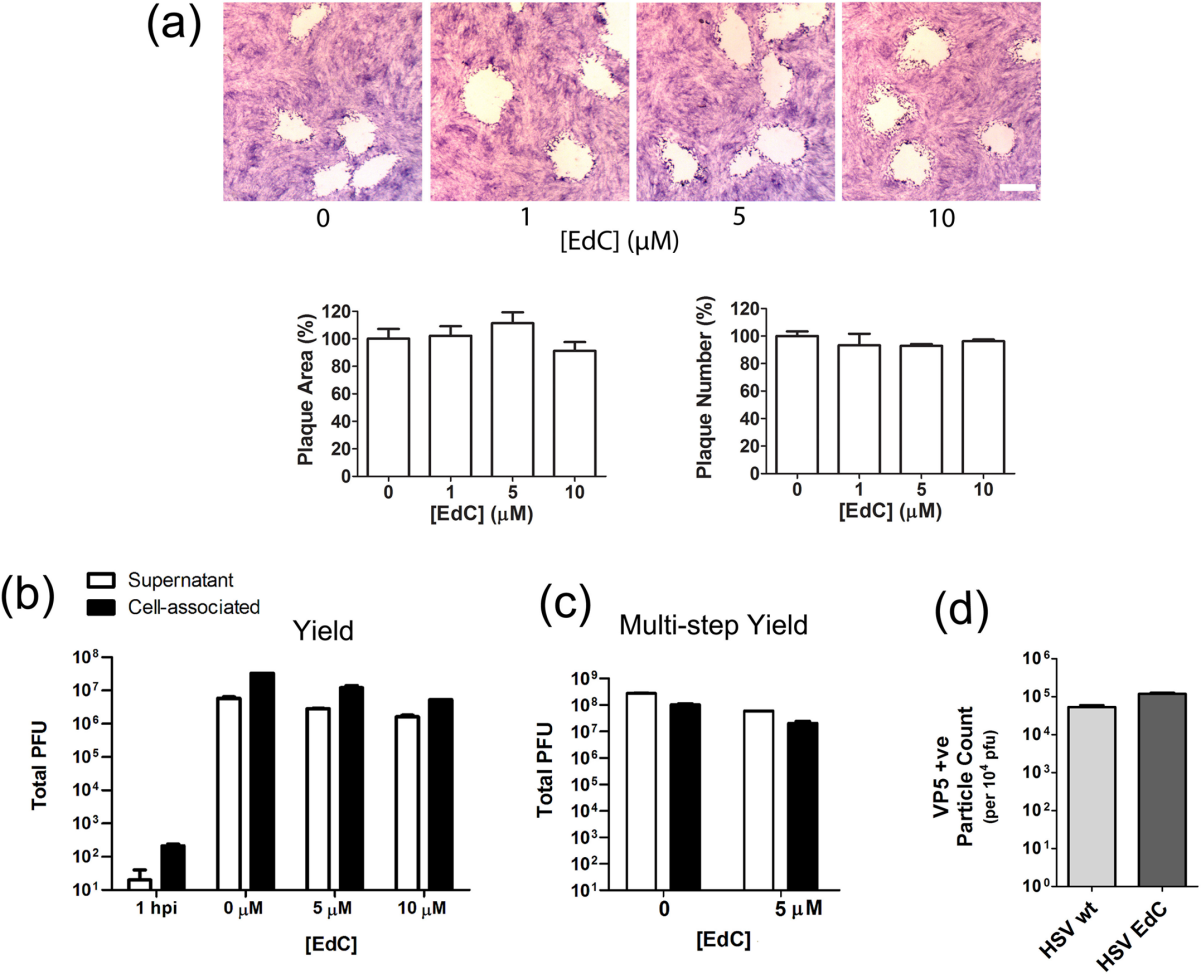
Minimal effect of EdC on HSV-1 replication. (a) RPE-1 cell monolayers were infected with 50 pfu of HSV-1[17] and incubated in the presence of EdC at various concentrations (added at 2 hpi). Plaques were fixed and stained at 48 hr (scale bar 1 mm). Plaque area (approximately 40 plaques) and plaque numbers at each EdC concentration were quantitated relative to untreated cells (set to 100%). (b) Single-step growth yield assay of HSV-1[17] in the presence of EdC. Cells were infected (moi 5) and incubated with EdC (added at 2 hpi). Supernatant and cell-associated virus was harvested at 20 hpi and titrated on RPE-1 cells. (c) Multi-step growth yield assay. Cells were infected (moi 0.005) and incubated with EdC added at 2 hpi. Virus was harvested at 72 hpi and titrated on RPE-1 cells. (d) Particles/pfu ratios of HSV and HSV^EdC^. Virus released into the medium and purified by ultracentrifugation was titrated and equal pfu applied to a defined area on coverslips and stained for VP5+ve particles. Multiple fields were imaged and tiled so that all particles in the samples were quantified. The graph indicates total particle counts from the accumulated individual fields and the SD of particle counts within individual fields.

Previous work from our own [24] and other laboratories [16, 25] has demonstrated the incorporation of alkyne-derivatised nucleotides into HSV replication compartments and the co-localisation of nascent replicating DNA with virus replication proteins using combined click-chemistry and immunofluorescence approaches. We found that EdC (ethynyl-deoxycytidine) was more sensitive for detection of HSV replication centres than EdU (ethynyl-deoxyuridine), consistent with the high GC content of HSV DNA [24]. In this work, optimising conditions for the production of HSV^EdC^ we found no significant effect of EdC even over relatively prolonged times (72 hrs) on uninfected cell growth or morphology (S1 Fig) and no significant effect on the efficiency of virus plaque formation nor plaque spread (Fig 1A), consistent with previous data [24]. In analysis of both single step (Fig 1B) and multi-step replication (Fig 1C), we found at most a minor effect (3–4–fold reduction) in overall yields. The particle/pfu ratio of HSV^EdC^ produced from multi-round virus replication and normal HSV produced in the absence of precursor were compared by particle counts using normalised amounts of pfu. The results show a modest increase for HSV^EdC^ (Fig 1D). We do not know the precise explanation for this slight increase though the value was within the range of particle/pfu we have found for normal HSV stocks (5–20). Overall even prolonged incubation with EdC at concentrations at least up to 10 μM was well tolerated, with minimal effect of virus replication and the progression of infection.

**Fig 2:**
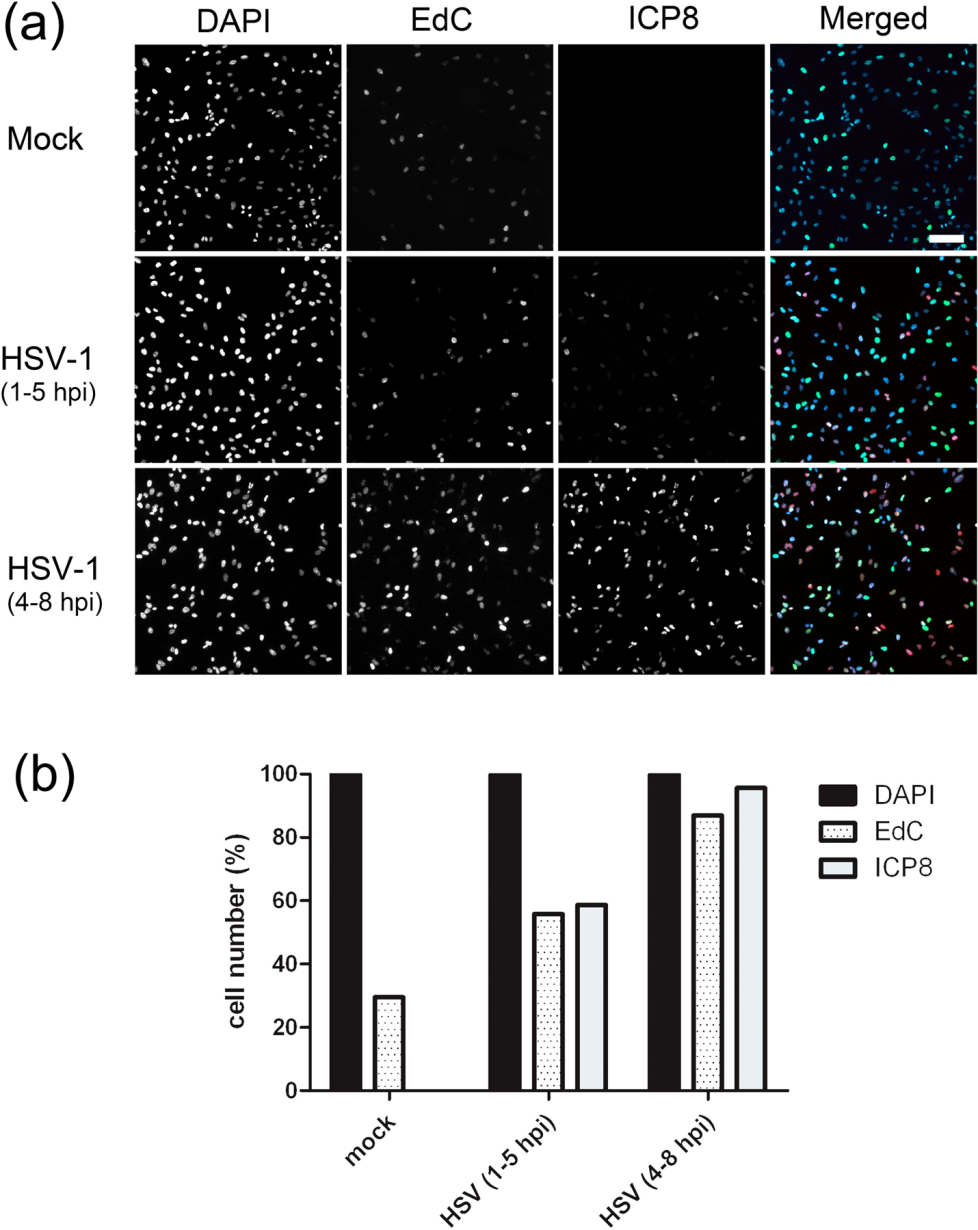
Temporal incorporation of EdC into viral replication compartments. (a) RPE-1 cells were infected (moi 10) and pulsed with 5 μM EdC for 4 hrs at the times indicated. Cells were fixed and processed for EdC incorporation together with immunofluorescence for ICP8 and counterstained with DAPI staining for total DNA (DAPI). Individual channels are shown in grey scale and the merged images in colour. (b) Using the DAPI channel as a mask, images were then quantified for EdC or ICP8 and the percentage +ve plotted against total cell nuclei count. Images were taken at 10x magnification (scale bar 100 μm).

EdC incorporation in uninfected cells (4 hrs) showed DNA synthesis in approximately 25–30% of cells (Fig 2A and 2B) with the spatial localisation patterns observed at higher magnification (S2 Fig) varying from discrete small clusters (S2i Fig) to more intense incorporation in prominent large focal clusters (S2ii and S2iii Fig) or homogeneous diffuse patterns (S2iv Fig). These patterns are consistent with previous spatial analysis of cellular DNA synthesis and reflect approximate stage within S-phase [20, 22]. In HSV infected cells, the percentage of positive cells increased to approximately 55% by 5 hpi (hours post infection) and virtually all cells were positive for EdC incorporation by 8 hpi (Fig 2A, summarised panel b).

**Fig 3:**
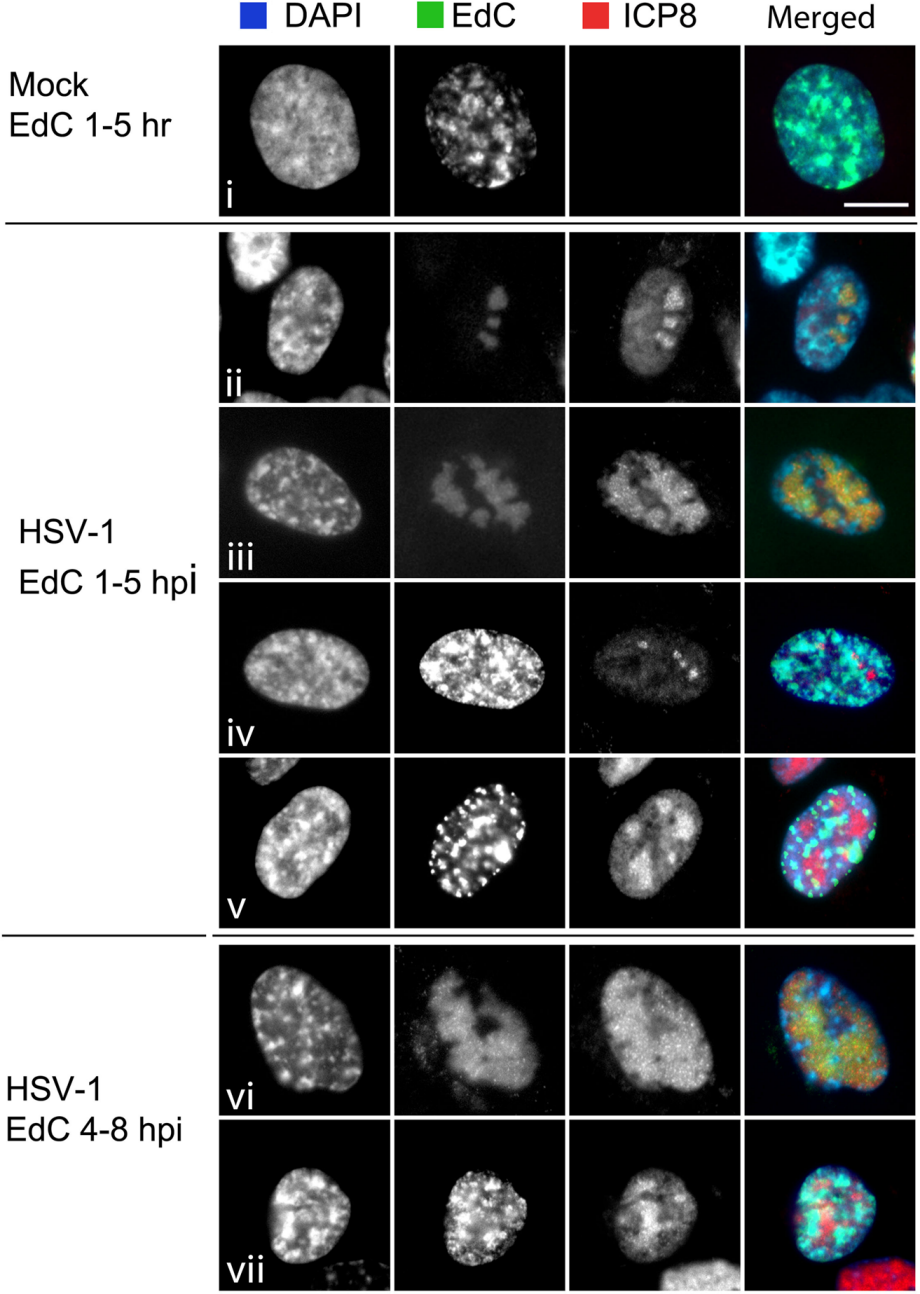
Distinct patterns of EdC incorporation in viral replication compartments. Higher magnification images from experiment as described for Fig 2. Cells were processed by cycloaddition for EdC incorporation together with immunofluorescence for ICP8 and counterstained by DAPI staining for total DNA (DAPI). Individual channels are shown in grey scale and the merged images in colour. Images were acquired with an x63 objective (scale bar 10 μm). Patterns of localisation are as discussed in the text.

From higher resolution spatial analysis of active DNA synthesis and localisation of the major DNA replication protein ICP8, several distinct patterns were observed (Fig 3). By 5 hpi, in those cells positive for DNA synthesis, the majority of cells showed discrete replication foci, colocalising with ICP8 (Fig 3, panels ii, iii) and reflecting the previously documented features of HSV DNA replication compartments [28–30]. We also observed populations of infected cells wherein multiple intense focal clusters of nascent DNA synthesis were observed, but in this case without any clear colocalisation with ICP8, which nevertheless still formed in distinct smaller (Fig 3, iv) or larger (Fig 3, v,vii) lobules. These latter patterns likely represent infection of cells which were in S-phase (or committed to S-phase and not prevented from doing so). The localisation of ICP8 likely represents some level of ongoing viral DNA synthesis in such S-phase cells, in the background of prominent cellular DNA synthesis. Again these results are entirely consistent with previous data [31].

### Production and genome detection in HSV virions containing EdC

We carried out a series of experiments to optimise conditions for the production of HSV^EdC^ and then scaled up (see materials and methods) with multi-round infection initiated at low multiplicity of infection (moi; 0.005 pfu/cell) and two pulse-labelling intervals in the presence of 5 μM EdC. Overall yields of HSV^EdC^ virus produced were virtually unchanged from normal virus production.

The lack of any pronounced effect of EdC on HSV yield and its incorporation into replication compartments does not necessarily mean that it will be efficiently incorporated or detectable in capsids. Therefore to examine the efficiency of detection of genomes in capsids we exploited an in vitro assay reported by Newcomb et al., [26, 27] which showed that HSV capsids, after absorption onto solid surfaces, underwent some form of structural rearrangement(s) such that with moderately elevated temperatures, genome expulsion from the capsids could be observed. Although the precise mechanism was unknown, these observations indicate that attachment to a solid surface perturbs the capsid, (or transmits a structural change to the portal) facilitating DNA release.

**Fig 4:**
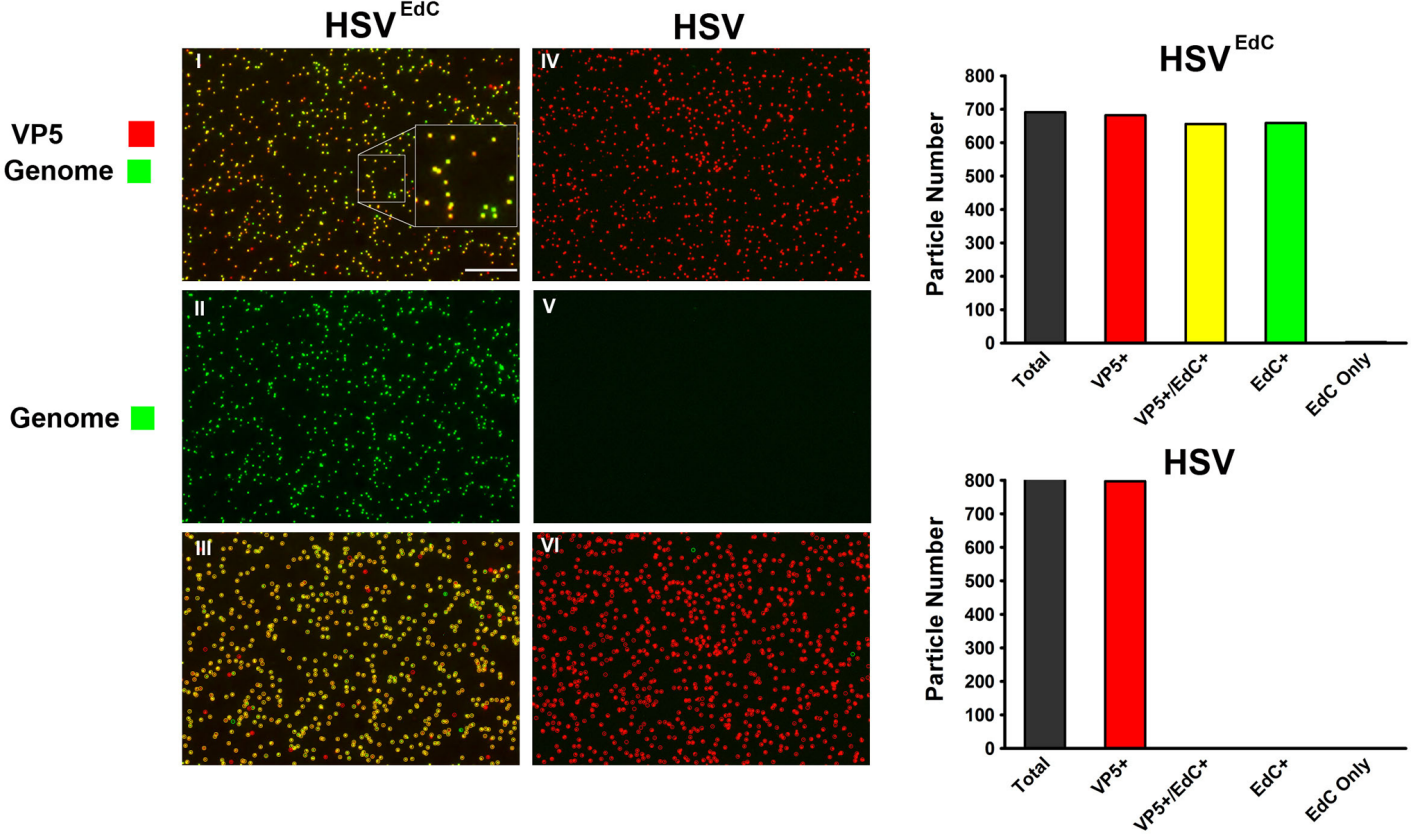
Quantitation of EdC labelled genomes in individual HSV-1^EdC^ particles. Equivalent samples of HSV-1^EdC^ or HSV-1[17] at 1x10^8^ pfu/ml were adsorbed onto glass coverslips prior to detection by cycloaddition and immunofluorescence for VP5. Panels I and IV show the merged channel images for each virus (scale bar 10 μm). The inset in panel I shows a magnified section. Panels II and V show only the green channel (genome detection) for each virus. Panels III and VI show the colour-coded outline overlay produced by the ImageJ plugin used for particle analysis (described in materials and methods); yellow indicates particles containing both VP5 capsid protein and EdC genome signal; red indicates VP5+ve particles lacking detectable EdC; green indicates particles with detectable EdC but no VP5. The data for approximately 700 particles are quantified in the right panels for each virus.

Samples of HSV^EdC^ or unlabelled HSV-1[17] were adsorbed onto borosilicate coverslips, fixed and processed for the simultaneous detection of genomes (green channel) and capsids (using anti-VP5 antibody, red channel). Typical results showing the merged image for HSV^EdC^ and HSV-1[17] are illustrated in Fig 4, panels I and IV respectively. The individual channels for genome detection are shown in corresponding panels II and V for each virus. Using an ImageJ plugin, capsids were enumerated and the signals quantitated in each channel (see materials and methods). Particles are categorised as a positive red particle or positive green particle, requiring positive particles to be not only above the background ROI but 1 standard deviation (SD) above the background ROI. The macro produces a colour-coded overlay (panels III and VI) in which particles containing both signals are coded yellow, particles that are capsid positive but lacking a genome signal above threshold are indicated in red, and particles with a genome signal but lacking a capsid signal are coloured in green. Quantitation is shown in the right-hand panels. Approximately 700 HSV^EdC^ capsid particles were identified in this representative field of which 95% were positive for genome detection. The small percentage of particles that do not contain a genome signal above threshold could be due to low detection signal, low EdC incorporation, defective particles, or genome release (see below). The extremely small numbers of green particles that were not detectable by VP5 immunofluorescence could also be due to defective particles or released DNA. However clearly the vast majority of HSV^EdC^ capsids contained detectable EdC containing genomes. The control HSV-1[17] had essentially no detectable genomes above background (Fig 4, IV-VI and panel), demonstrating the extreme specificity of the reaction. From our analysis, the percentage of HSV^EdC^ particles with detectable genomes was comparable if not slightly greater than that for similarly labelled Adv (using a combination of EdA and EdC), where approximately 90% of capsids applied to coverslips contained detectable genomes [16]. This information is important since if incorporation efficiency was low, e.g., only 10% of particles were detected or the efficiency was unknown, then subsequent studies examining genome localisation may give an incomplete picture.

Further quantitation of HSV^EdC^ is given in S3 Fig, including the comparative distributions and variance for VP5 detected by immunofluorescence and genomes detected by cycloaddition. Gaussian distributions were fitted to each channels’ frequency data using Image J curve fitter. We used the coefficient of variation (CV, (σ/μ) x 100) as a measure of variance. The goodness of fit to a normal distribution of VP5 intensities exceeded 0.95 with a CV of 23.75 (S3 Fig). This variance in particle intensity is very similar to analogous types of HSV single particle analysis using either GFP-fusion proteins or antibody to capsid protein [32, 33]. With this as a benchmark, we found the distribution genome signal detected by cycloaddition to have only marginally increased variance with a CV of 34.19 and a goodness of fit to normal of 0.94 (S3 Fig).

The scatter plot for individual particle analysis is illustrated in S4 Fig for both HSV^EdC^ and HSV, showing the vast majority of genome-positive particles with only low numbers of particles having a genome signal below threshold. HSV w/t exhibited a single focus above background, likely an artefact of detection. Taken altogether, these results demonstrate that EdC is efficiently incorporated into HSV replication compartments and subsequently into genomes in mature HSV particles, that the yields and infectivity of such particles are minimally affected and that the genomes of the majority of such particles can be detected in vitro by cycloaddition reaction.

### Capsid ejection and detection of genomes by cycloaddition

Our results by definition detect HSV^EdC^ genomes by cycloaddition after virions are adsorbed onto coverslips. We also found in additional control experiments that the vast majority of HSV^EdC^ genomes (99%) were not detectable if the cycloaddition reaction was performed on virions in physiological buffer, prior to adsorption to the coverslips (S5A and S5B Fig).

In the original observations using purified capsids, Newcomb and colleagues observed that DNA was realised as elongated strands with progressively increasing ejection at elevated temperatures [26, 27]. Although there was considerable heterogeneity between particles, release could be substantially prevented if the capsids were first cross-linked with PFA. We detected genomes within virions as punctate foci, but we did not observe elongated genome release. However our analysis was on extracellular virions and not capsids. To examine the detection and possible ejection of HSV^EdC^ genomes further, we analysed virions that had been adsorbed onto coverslips and then subject to elevated heat treatment. The results were striking (S5C Fig). Whereas adsorption of virions at room temperature resulted in detection of genomes colocalised within capsids (e.g. panel a, also Fig 4), elevated temperature resulted in numerous elongated filamentous stands ejected from virions (panel c). This was accompanied by an increase in the numbers of capsids in which genomes were not detected as well as an increase in the numbers of punctate genome foci that were not detected by immunofluorescence. Our results are entirely consistent with the previous data obtained by electron microscopy and demonstrate that HSV^EdC^ genomes when released from heat-disrupted virions could readily be detected on coverslips by the cycloaddition reaction. Furthermore, they indicate that when absorbed onto solid supports at lower temperatures, the genome is available for the cycloaddition reaction, presumably due to some conformational perturbation of the virion/capsid, but maintained in the confines of the capsid or virion.

### Genome trafficking and nuclear transport

**Fig 5:**
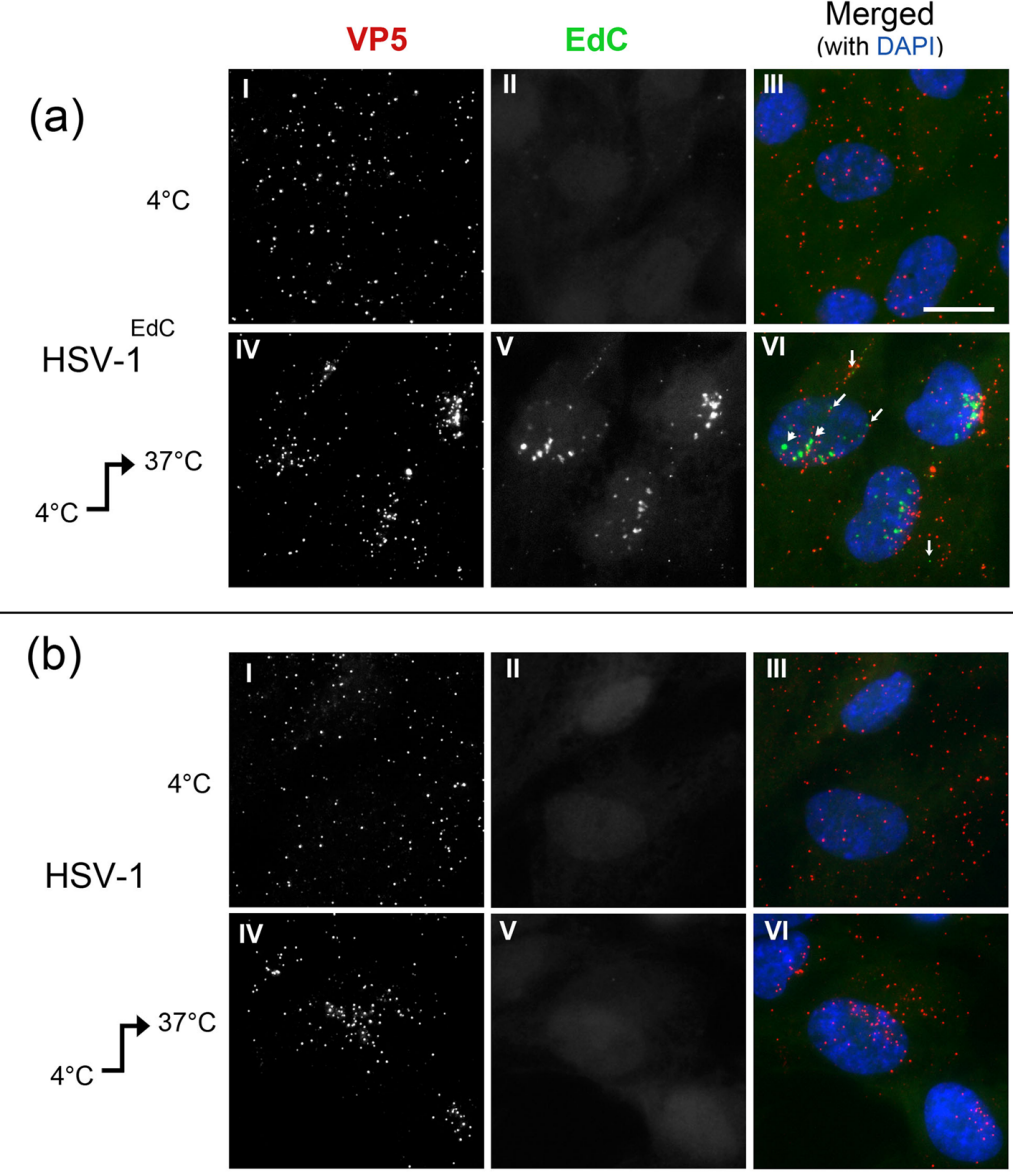
HSV-1^EdC^ genomes detected only after cell entry and uncoating. Cells were infected with (a) HSV-1^EdC^ or (b) HSV-1[17] at moi 10 at 4°C and incubated for 45 min. Cells were then either fixed immediately (4°C), or the temperature was raised to 37°C for 2 hr (4°C → 37°C). Genomes were detected by cycloaddition and capsids by anti-VP5 immunofluorescence (scale bar 10 μm). In each of (a) and (b), panels I and IV show detection of VP5, II and V show detection of EdC labelled genomes, with panels III and VI show the merged image.

We next investigated HSV^EdC^ genome transport and uncoating in cells in vivo. Virus (moi 10) was adsorbed onto cells at 4°C for 45 min and then either washed and processed directly or shifted to 37°C to allow fusion and virus entry and then processed 2 hrs later (Fig 5). At 4°C for both HSV^EdC^ and HSV (Fig 5A and 5B respectively), numerous virus particles could be detected on cells (VP5, panels I). In contrast to adsorption onto coverslips, there was no significant genome signal for HSV^EdC^ particles adsorbed onto cells (Fig 5A, 4°C, EdC channel, panel II). This is consistent with the lack of genome detection in physiological buffer and supports the proposal from this and previous work [26, 27] for a conformational perturbation upon adsorption to artificial surfaces which is registered by the cycloaddition reaction. After shift to 37°C for 2 hrs, numerous capsids could be detected within the cytoplasm but with only infrequent detection of genomes (Fig 5A, panels IV-VI, see also below). In contrast, distinct genome foci were now readily observed in the nucleus (Fig 5A, panels V and merged VI). We frequently observed smaller genome foci in close proximity to capsids (panel VI, small angled arrows) together with larger nuclear foci (arrowheads). Low but detectable numbers of foci could be observed in a minority of cells in the cytoplasm (e.g., panel VI, vertical arrows). The HSV^EdC^ foci detected within the nucleus were somewhat heterogeneous in size at this time (2 hrs) and distinctly larger than the few foci detected within the cytoplasm (see below). No specific genome signal was observed for the control HSV-1[17] (Fig 5B, panels V-VI). In further control experiments the appearance of nuclear genome foci after HSV^EdC^ infection was completely dependent upon the copper-catalysed cycloaddition reaction, was blocked by incubation with neutralising antibody prior to infection and was not prevented by prior treatment of HSV^EdC^ virions with DNAse (S6 Fig).

Altogether these data provide robust support for the proposal that the nuclear foci represent uncoated HSV^EdC^ genomes that have reached nuclear pores, uncoated and have been transported into the nucleus, with infrequent (though detectable) presence in the cytoplasm of some cells.

### Temporal alteration in genome compaction state

**Fig 6:**
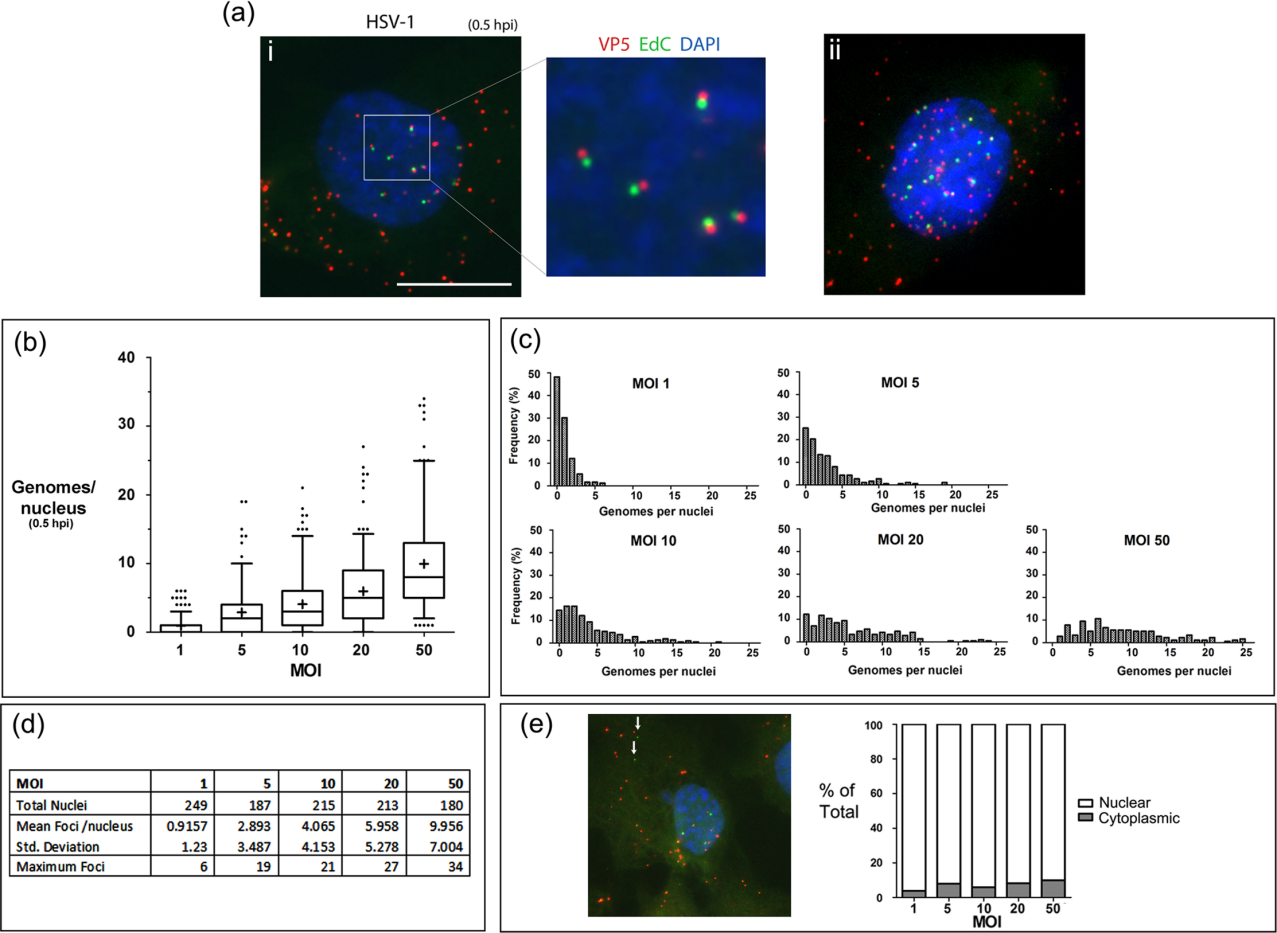
Quantitative analysis of genome uncoating at 0.5 hpi. (ai) Representative high magnification image of an individual cell infected with HSV-1^EdC^ (moi 10) at 0.5 hpi. Infection was as described in Fig 5. The expanded inset shows juxtaposition of uncoated compact genomes (green) and parent capsid (red) (scale bar for main image 10 μm). An example of a nucleus containing more numerous genomes is shown in panel ii. Distributions frequencies of genome numbers for approximately 200 nuclei is shown in panel (b), representing a box and whisker plot for genome number per cell nuclei at 0.5 hpi at increasing moi. Box limits represent 2^nd^ and 3^rd^ quartiles with the horizontal bar in the middle showing the median and whiskers showing up the 5–95% range of the total population. Exceptional outliers (less than 5% of population) are shown as individual dots. The mean value is indicated by a ‘+’. Raw data for this summary is shown in the panel (d) below. (c) Histograms for number of genomes observed in cell nuclei at 0.5 hpi at each moi. Bin width was set at 1 genome and approximately 200 nuclei for each moi were analysed for (b) and (c). (e) Distribution of total labelled foci seen in the cytoplasm versus the nucleus at each moi.

We next undertook a quantitative examination (Fig 6) of the relationship of moi to the numbers of genomic foci, evaluated at 30 min after shift to 37°C, in this case co-staining with anti-VP5 to detect infecting capsids. We used 30 min to quantitate the relationship with moi since foci became more difficult to quantitate accurately at later times because of the changing morphology at 2–3 hpi (see below) and because immediate early protein synthesis had almost invariably already occurred by 2 hpi. Fig 6A panel I shows a representative maximum projection image of cells 30 min after HSV^EdC^ infection, illustrating the frequent occurrence of genomes at a consistent and close proximity to capsids, most likely representing recent uncoating events where the genome had not yet physically moved far from the capsids from which they emanated (Fig 6A, inset). At this early stage, HSV^EdC^ nuclear genomic foci were comparatively homogeneous in size and shape and generally spherical. Mean numbers of genomes per nucleus observed at different mois (approximately 200 cells at each moi) are illustrated in Fig 6B (summarised in panel d), with frequency distributions indicated in Fig 6C. With increasing moi there was an increasing trend of genome foci in the nucleus, but this was not directly proportionate, with for example an average of 4–5 foci at moi 10, 6 at moi 20 and 10 at moi 50. Moreover, while representing minority populations high numbers of foci could be observed in some nuclei (example, panel a, ii) with maximum numbers for moi 10, 20, and 50 being 21, 27 and 34 respectively (Fig 6B and 6D). Later in infection while there was a trend of increased numbers of nuclear foci, this was generally less than a 50% increase but more difficult to quantitate as indicated above. Capsid-free cytoplasmic foci (example, Fig 6E, vertical arrows) were infrequently observed though at higher mois these could represent 7–8% of the total foci (Fig 6E). While these were a minor population, such cells could be relevant e.g., to overall cellular responses (see discussion).

Standard wide field microscopy is limited by diffraction and additional inherent limitations in optical imaging and capture. To examine the genomes in more detail, we pursued super-resolution microscopy using 3D structured illumination microscopy (3D-SIM)[34].

**Fig 7:**
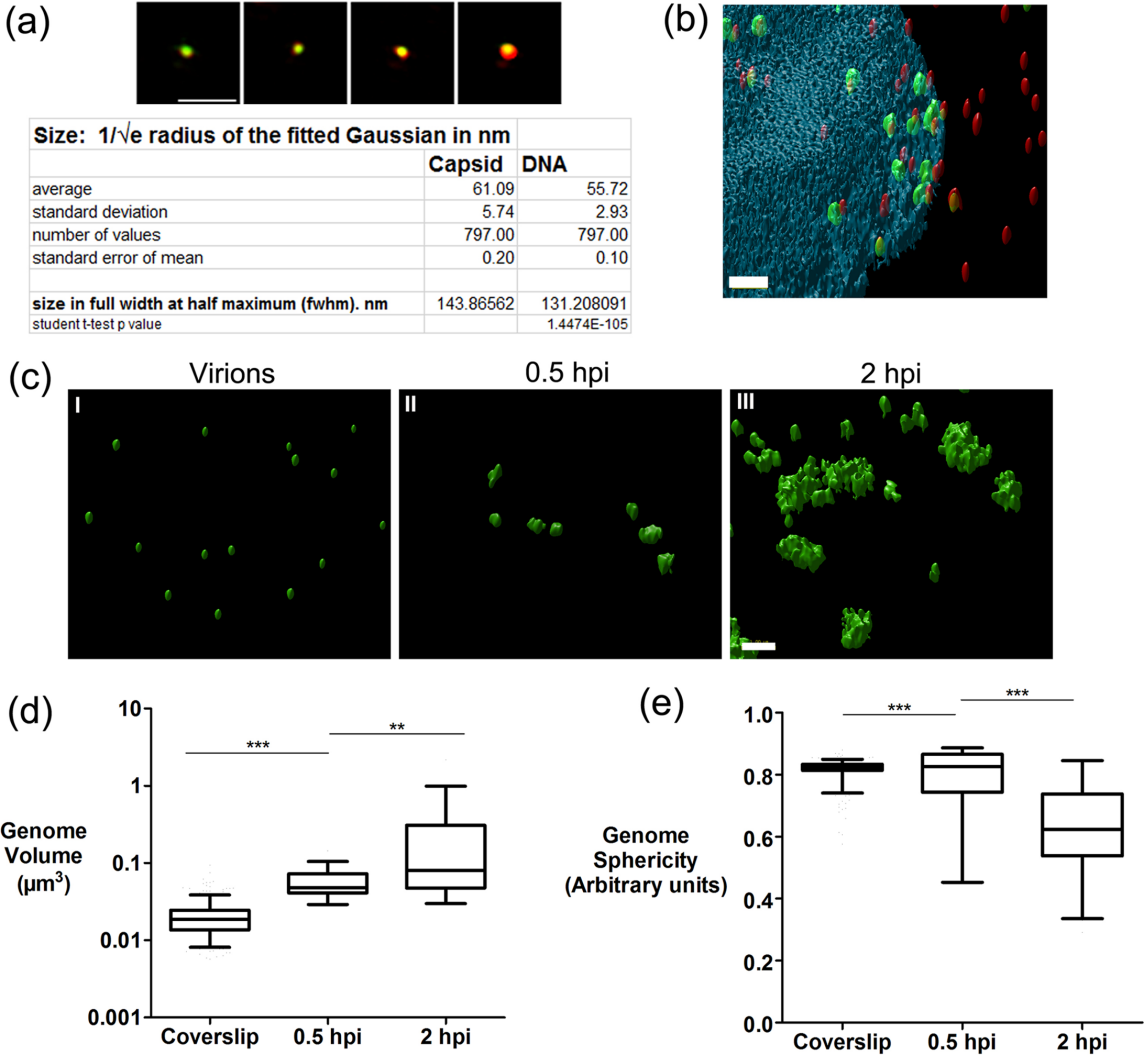
3D-SIM analysis of genome decompaction. 3D-SIM data of HSV^EdC^ virions adsorbed to glass coverslips as described in Fig 4. (a) Raw data was reconstructed and individual representative particles are shown as Z-projections. Quantitative analysis was carried out on approximately 800 particles via 2D-Gaussian fitting to calculate full width half maxima in each channel with numerical summary data given in the panel. (b) 3D-SIM data of a cell infected with HSV-1^EdC^ (moi 20) and examined at 0.5 hpi. Raw data was visualised by iso-rendering in Huygens analysis software as described in materials and methods (scale bar 1 μm). Red objects denote VP5 capsids, while green objects denote EdC-labelled genomes. Blue object is nuclear DAPI staining. (c) 3D-SIM data of HSV-1^EdC^ genomes on coverslips compared to infected cells at 0.5 hpi and 2 hpi visualised after 3D-SIM by iso-rendering in Huygens analysis software (scale bar 1 μm). Quantitative analysis of genome volume (d) and sphericity (e) is shown as box and whisker plots. Boxes show 2^nd^ and 3^rd^ quartiles with a horizontal bar in the middle showing the median, while whiskers show up to 5–95% of the total population. 50 genomes were analysed for each category. Unpaired two-tailed t-tests were used for statistical results (** = p<0.005, *** = p<0.0001).

We first extended the analysis of virions on coverslips combining capsid and genome detection followed by 3D-SIM, as described in materials and methods. Raw data (five phases, three angles per plane) was then computationally reconstructed and representative individual particles from a maximum intensity projection of the 3D data is shown in Fig 7A. Further particle analysis (approximately 800 particles) was performed via 2D-Gaussian fitting in each channel to calculate dimensions at full width half maxima (FWHM, summarised in table of Fig 7A). For the capsid and genome signals the mean FWHM were 144 nm and 131 nm respectively. Although the mean genome signal was slightly smaller than the mean capsid signal, we do not take this as a significant difference between capsid dimension and packaged genome dimensions, the resolution of which is beyond the limits of these techniques. On the other hand 3D-SIM combined with quantitative object analysis allowed a better resolved spatiotemporal analysis of nuclear entry in the cell.

We examined genome presentation in the nucleus at early times of infection (0.5 hpi). Cells were imaged by 3D-SIM and the data processed using the Object analyser module of Huygens image processing software in which after 3D segmentation, geometrical and spatial localisation data can be calculated for individual objects. A representative 2D field of the 3D rendered image is shown in Fig 7B (tilted to reveal the z-dimension). This field shows capsids in the red channel, genomes in green and DAPI stained nucleus in blue, with transparencies applied. An accompanying 3D video animation is shown in S7 Fig. The inherent lower optical resolution in the z-dimension than x/y-dimensions results in slightly oblong capsids. This optical limitation applies also to the genomic foci but does not affect the main conclusions on comparative volume between genomes. We compared virions on coverslips versus infected cell nuclear genomic foci at 0.5 hpi and 2 hpi (Fig 7C). For clarity in the infected cell nuclei only the genome signal is shown. The 3D genome objects were quantified for volume (Fig 7D) and shape (Fig 7E), the latter measured as proximity to a sphere (sphericity). The results demonstrate a substantial increase (approximately 3-fold) in mean volume of the nuclear genomic foci compared to those in virions (Fig 7C panels I, II; Fig 7D). Nuclear foci at 0.5 hpi were comparatively homogeneous and with only marginal differences in sphericity (Fig 7D and 7E). The linear length of the HSV genome is approximately 50 μm and would stretch across a typical cell several times. Our results demonstrate that while HSV genomes clearly expand, they are initially constrained and condensed to a comparatively consistent compact, roughly spherical volume. By 2 hpi, the mean volume of the foci had increased further (by another 2–4 fold), but there was also a marked increase in irregularity as the foci became more decondensed and dissipated. As infection progressed beyond 2 hrs, the genome signal became more difficult to quantitate evolving into diffuse, dissipated aggregates of lower intensity, but frequently with smaller, more condensed foci remaining (see below), usually on the perimeter of the decondensed material.

**Fig 8:**
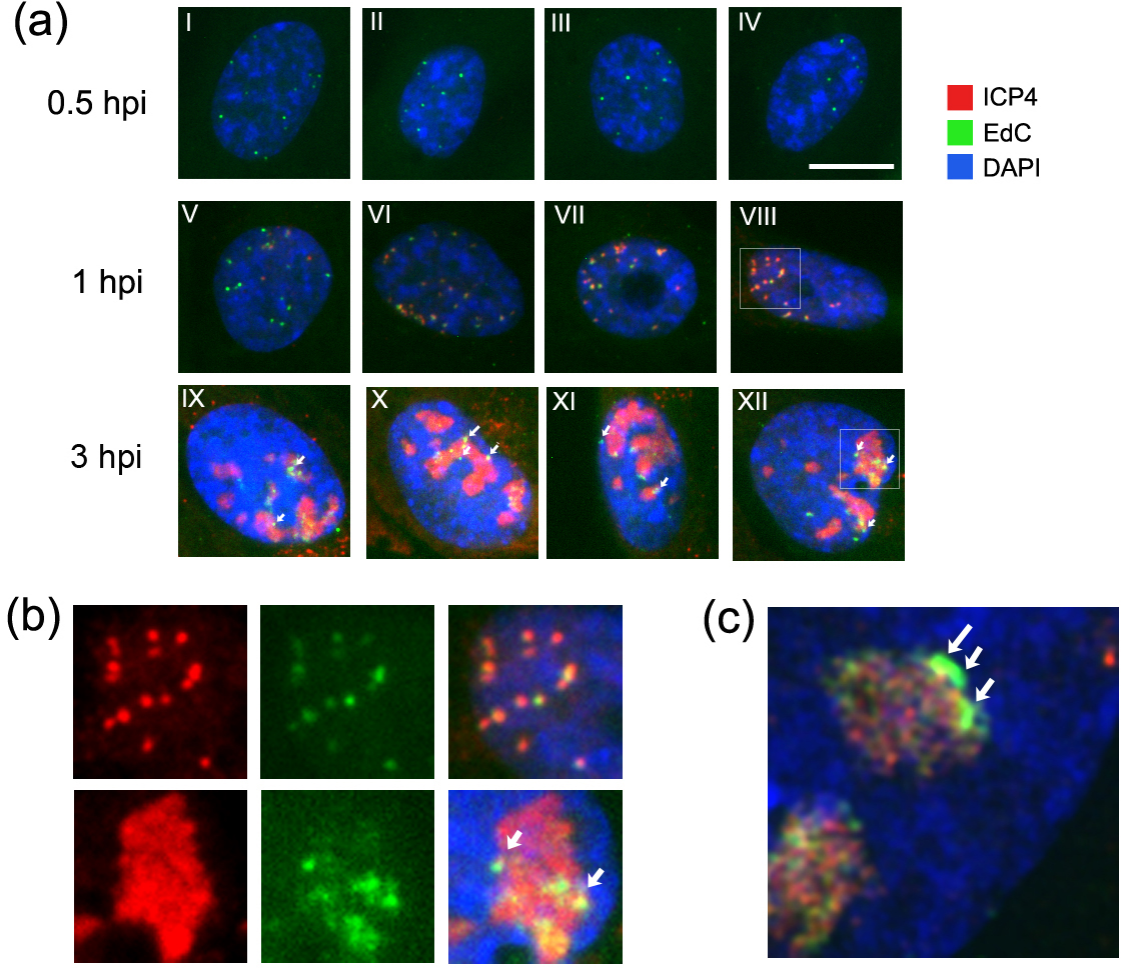
Spatiotemporal relationship of genome decompaction and ICP4 expression. (a) Representative images of cells infected with HSV-1^EdC^ (moi 10). Infection was synchronised as described in Fig 5 and cells fixed at 0.5 hpi (I-IV), 1 hpi (V-VIII), or 3 hpi (IX-XII) with subsequent detection by cycloaddition and immunofluorescence for ICP4 (scale bar 10 μm). Insets from panels VIII and XII are shown magnified in (b) to illustrate a shift from ICP4 association with genome foci immediately after infection but reduced or absence of association on foci remaining at the later times. (c) 3D-SIM data of a cell nucleus infected with HSV-1^EdC^ and fixed at 2 hpi showing residual EdC labelled infecting genomes remaining as tighter foci on the periphery of replication compartments, marked by ICP4 (red) and the absence of significant ICP4 recruitment to those remaining genome foci.

To examine this alteration in genome compaction further we extended the analysis to investigate the relationship between localisation of ICP4, the major HSV IE regulator of transcription and genome morphology. Representative fields (four examples at each time point) illustrating several features are shown in Fig 8. As shown above, within 30 min after shift to 37°C, new discrete nuclear foci (green channel) were readily observed. These were detectable prior to the accumulation of ICP4 (Fig 8, 0.5 hpi). By 1 hr, together with the progressive increase in size of genome foci, as ICP4 became detectable we observed several patterns of localisation. These included cells with ICP4 recruitment to a subset of genomes with several foci still having undetectable levels (e.g., panel V); cells with most foci accumulating ICP4 at some level (panels VI-VII) and cells with virtual quantitative accumulation of ICP4 on all genomic foci (panel VIII). At this time there was comparatively little diffuse ICP4 with the majority co-located with genome foci. By 2–3 hrs a qualitative change was observed in this association. As discussed above, the infecting genomic foci appeared as increasingly heterogeneous dispersed aggregates of lower intensity, together with residual punctate foci frequently on the periphery of the dispersed pattern (Fig 8A, 3 hpi). However while ICP4 exhibited quite precise co-localisation with the genome foci at 1 hr, by 3 hrs ICP4 was observed more within the dispersed areas of genome labelling, lacking any distinct enrichment in the remaining punctate foci. This was a distinct qualitative feature wherein foci remaining at later times on the periphery of the diffuse areas either completely lacked or were not enriched in ICP4, while foci at earlier times selectively recruited ICP4. This distinction of ICP4 localisation on earlier condensed (1 hr, panel VIII) versus later more decondensed foci (3 hrs, panel XII) is shown in expanded form (Fig 8B) with each channel separate and merged. A separate analysis by 3D-SIM is shown in Fig 8C, (2 hpi), illustrating a more condensed genomic signal (arrowed) on the edge of extended decondensed area, with ICP4 localised preferentially within the decondensed material and virtually excluded from remaining punctate focus.

### Transcription and translation coupled transitions in infecting genome compaction state

**Fig 9:**
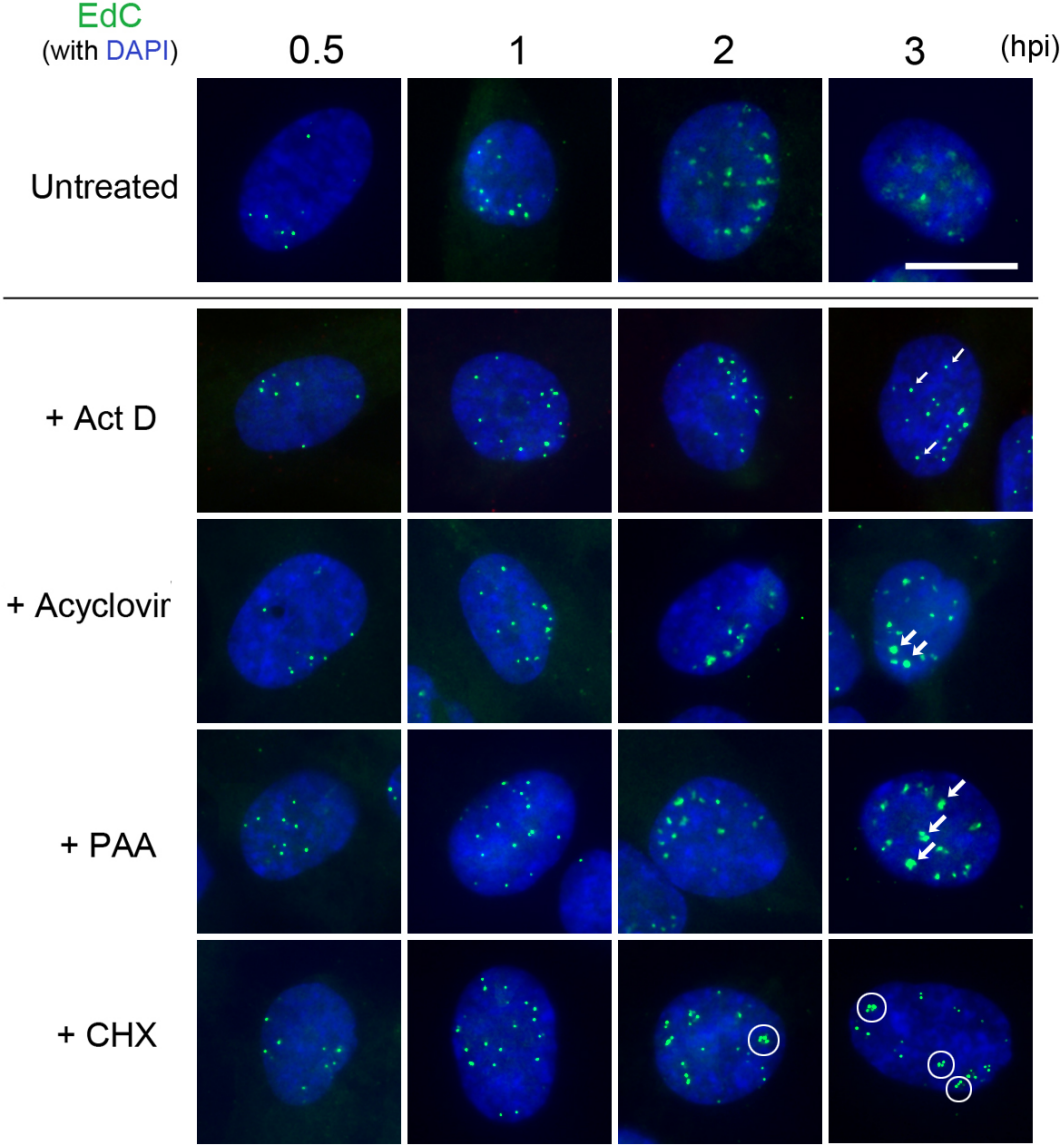
Effects of inhibition of transcription, translation and virus DNA replication on transitions in genome decompaction. Representative images of cells infected with HSV-1^EdC^ (moi 10) and incubated in the presence of ActD (5 μg/ml), ACV (500 μM), PAA (400 μg/ml), CHX (100 μg/ml), or no treatment. Infection was synchronised as described in Fig 5 and cells fixed at the time points indicated for processing (scale bar 10 μm). Arrows and circles indicate qualitative features of genome localisation under each condition as discussed in the text.

To examine the relationship between metabolic processes acting in the infected cell nucleus and virus genome localisation and compaction, we next investigated the effect of inhibition of transcription, viral DNA synthesis, or translation on genome localisation to the nucleus and the morphological condensation state. Cells were infected (moi 10) either untreated or in the presence of a series of inhibitors each added 1 hr prior to infection; actinomycin D (Act D, 5 μg/ml), acyclovir (ACV, 500 μM), phosphonoacetic acid (PAA, 400 μg/ml), or cycloheximide (CHX, 100 μg/ml) to inhibit transcription, virus DNA replication and protein synthesis. Control experiments confirmed activity of the drugs e.g., ICP4 protein synthesis was completely blocked by Act D and CHX but not by PAA or ACV (S8A Fig). Representative results for genome uncoating and morphology for each inhibitor at each of four early time points are shown in Fig 9.

In the absence of drug treatment, genome foci were observed in the nucleus by 0.5 hpi together with the temporal increase in volume, irregular morphology (1, 2 hpi), progressive decondensation and dissipation (3 hpi) described above. Two significant conclusions could be made from results of inhibition of transcription. Firstly there was no significant effect on either the average initial numbers or morphology of uncoated nuclear genome foci, indicating that transcription per se (for example, by a transcription-coupled ratcheting process) played no discernible role in genome uncoating and transport into the nucleus (Fig 9, + Act D). Secondly however it was clear that inhibition of transcription did have a significant effect on the progressive increase in genome volume and eventual decompaction, which were almost completely inhibited (cf, 3 hpi, untreated versus +Act D).

**Fig 10:**
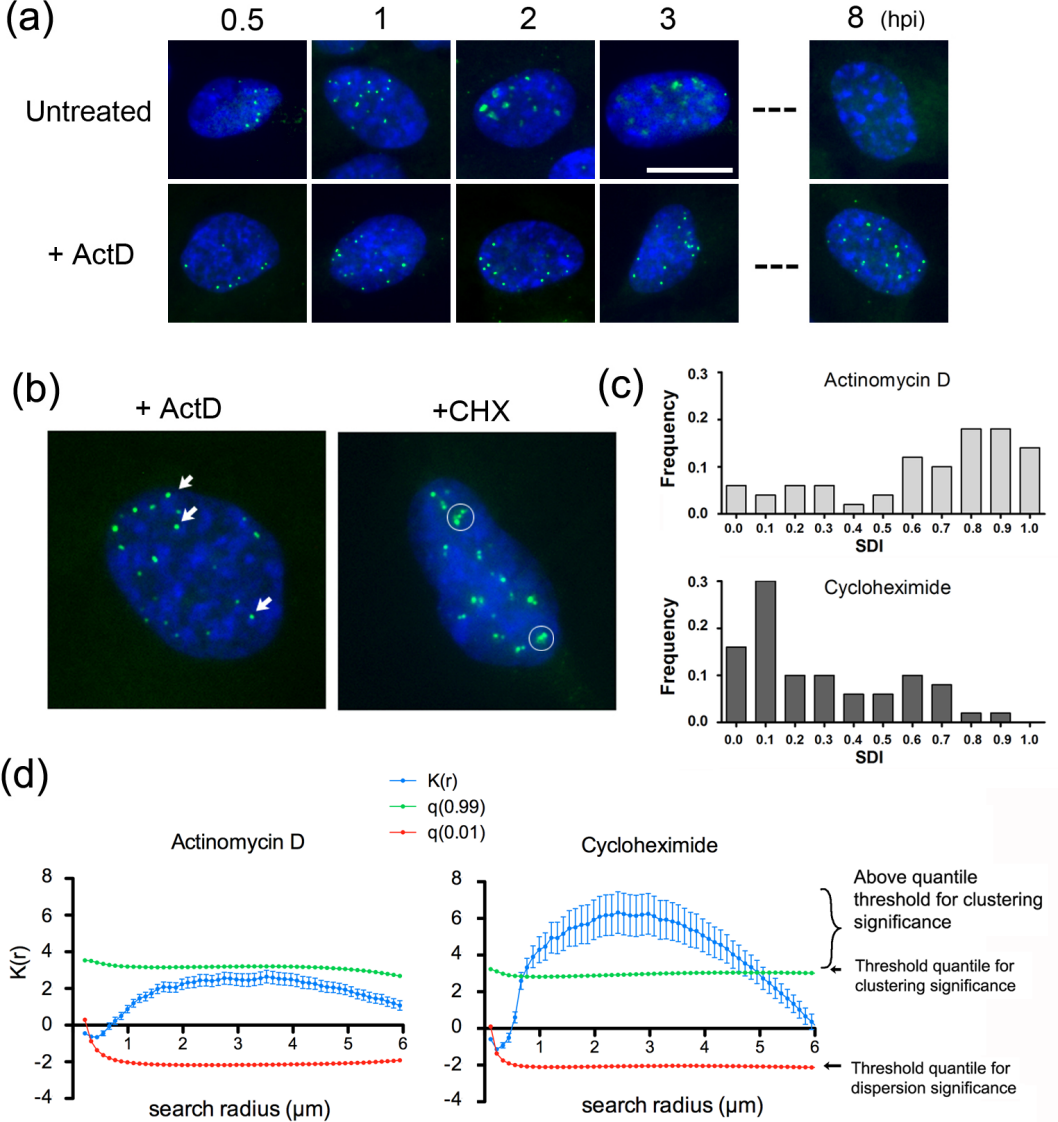
Comparison of the effects of inhibition of transcription versus translation on infecting genome localisation. (a) Infection and analysis as for Fig 9 in this case extended until 8 hpi, revealing the maintenance of tight condensed foci in the presence of Act D. (b) Comparison of genome localisation at 3 hpi in Act D treated versus CHX treated cells. Circles indicate the feature of genome clustering seen in CHX treated cell as opposed to the more typical individual foci (arrowed) for Act D. (c) The SDI distribution among populations of Act D and CHX treated cells were calculated as discussed in the text and materials and methods. SDIs close to 1 indicate a tendency to dispersion while closer to 0 indicates clustering. The differences in SDI frequency distributions between Act D and CHX were highly significant with that of CHX reflecting a clear trend to clustering. Histograms of the SDI were calculated from 50 nuclei from each group and the difference in distributions calculated using the Kolmogorov-Smirnoff test (p < 0.0001, D = 0.52). (d) Independent estimation of clustering by calculation of the K-function at increasing length of test radii. Data shown are the mean +/- sem of the K function for radii between 0.11μm and 6 μm, with corresponding low and high quantiles (0.01 and 0.99 respectively) for 47 cells treated with Act D and 52 cells treated with CHX. The tendency towards clustering is highly significant for the CHX treated cells.

These results were distinct from those obtained after inhibition of virus DNA synthesis. Similar numbers of uncoated infecting genome foci were initially observed, not unexpectedly. In this case however, a progressive increase in foci volume was observed, with a clear difference especially by 3 hpi for the ACV/PAA treated cells versus the Act D treated cells (cf, small arrowed foci in Act D versus larger foci in PAA/ACV panels). On the other hand, inhibition of DNA replication clearly had an effect, preventing the later dissipation and decrease in intensity seen in untreated cells (cf, 3 hpi, untreated versus ACV/PAA), indicating that these latter events were likely related to genome replication or other coupled events. The smaller genome foci size and tighter morphology in the presence of Act D compared to that observed in the presence of ACV or PAA also indicates that events linked to or downstream of transcription per se, but not requiring DNA replication, are reflected in increasing volume and irregularity and decreased compaction of the uncoated genomic foci. We repeated this analysis for Act D including relatively late times (8 hpi), comparing the fate of infecting virus genomes during normal progression of infection or when transcription was inhibited (Fig 10A). Under normal conditions, the temporal trend to increasing dissipation and decreased signal intensity continued so that by 8 hpi, the diffuse dispersed signal from incoming genomes was extremely low (Fig 10A). In distinct contrast, in the presence of Act D, not only were genomes maintained in more condensed foci, they were maintained until at least 8 hpi, with minimal changes in numbers or morphology (Fig 10A, + Act D).

We also examined genome localisation in the presence of cycloheximide. Abundant accumulated data has shown that, unlike in the presence of Act D, in the presence of CHX transcription occurs but, in the absence of HSV protein synthesis, is limited to IE loci with little or no DE transcription nor DNA replication. CHX did not block uncoating and the numbers of nuclear genome foci at 0.5 hpi were similar in CHX treated to untreated and to each of the other inhibitors (Fig 9, CHX). However, we observed a distinct feature in genome morphology at subsequent times with CHX treatment. Thus whereas inhibition of transcription resulted in the maintenance of tighter more condensed and largely singular foci (Figs 9 and 10A and 10B), genome localisation in the presence of CHX resulted in a distinct congregation and clustering of genomic foci (Fig 9, CHX, circled clusters). While individual singular foci could still be observed in CHX treated cells, this was a noticeable qualitative change in genome presentation especially at later times (see Fig 10B, comparison at 3 hpi of untreated, Act D treated and CHX treated).

To examine this difference in an unbiased quantitative manner, we used the spatial statistics and focus clustering algorithm in ImageJ [35]. This algorithm (see materials and methods) examines the positions of objects within a reference structure (nuclei in this case) and assesses clustering using a normalized measure of the difference between the observed distribution of inter-point nearest neighbour distances and a completely random one. This difference is termed the Spatial Distribution Index (SDI). S9 Fig shows a schematic illustration of the analysis of the SDI. Panel a illustrates theoretical nuclei with random, clustered or dispersed patterns. The algorithm compares the cumulative inter-point distance frequencies (CDFs) for a truly completely random distribution (panel b, black lines in each graph; 95% confidence limits in grey lines) with the actual cumulative distribution obtained for foci in each example pattern (panel b, red lines in each graph). An actual random distribution (left cell) will show a distribution overlapping the theoretical random distribution while clustered (middle cell) or dispersed (right cell) distributions deviate significantly to the left or right respectively (cf, red and black CDFs in each panel). SDIs are then calculated as a probability index with a SDI close to 0 indicating a more clustered pattern, while SDIs closer to 1 indicate a pattern that is more evenly spread out. The theoretical overall distributions of SDIs for populations of cells is then calculated (S9C Fig bottom panels). A truly random pattern will show approximately even distributions of SDI values between 0 and 1 while clustered SDI distributions will show a distinct leftward shift towards lower values and evenly spaced distributions show a shift towards 1. We validated this approach using the completely random pattern of spots when viruses were applied to coverslips (S9E Fig). The CDF function of these capsid foci (S9F Fig, red line) directly overlapped with the theoretical random distribution pattern for the image (black line). Note while clusters can be observed in the distributed capsids, such clusters will occur by definition, but there is no significant difference between the overall distribution and a random pattern (S9F Fig).

We applied this nearest neighbour analysis to virus genomes in infected cells for individual nuclei (approximately 50 nuclei, 3 hpi) in the presence of Act D or CHX. SDIs were calculated for the foci in each nucleus and compiled into a distribution of SDIs across the cells for each condition. Consistent with the indication from visual inspection (Figs 9 and 10B), the results indicate a clear difference in the population distribution of inter-foci distances in Act D versus CHX treated cells (Fig 10C). While the Act D pattern deviates from random to some degree across the population, this was bordering on statistical significance (p-value = 0.056; D statistic = 0.26). What was clear was the distinctly different trends for CHX versus Act D, indicating a highly significant change in relative localisation and clustering of genomes in the presence of CHX versus Act D (p-value < 0.0001; D statistic = 0.52).

In a second approach to support these conclusions we examined clustering by an independent method, the distance-based K function (Ripley function). In this method (see materials and methods) using the BioImage Analysis platform ICY [36], nuclei are segmented and EdC labelled genomes located similarly to the approach above. Regions of interest (circles) with increasing radii are drawn around each detected spot and other spots located within the circles are identified. The K-function, *K(r)*, is based on the average number of points inside a circle of radius *r*, calculated for the increasing radii and has an expectant value of zero for a random distribution of spots. The amplitude of the K-function can then be compared to corresponding low and high quantiles of completely random distributions (0.01 and 0.99 here). When *K(r)* is higher than the high quantile for a certain radius, the foci are significantly organised in clusters. Conversely, when K is lower than the low quantile, the foci are dispersed. The results were very clear and indicated a distinct statistical significance for clustering of genomes in CHX treated cells with search radii from 1–4 μm and a tailing off as the radii became too large to attribute significance (Fig 10D). In contrast the spatial distribution of genomes in Act D treated cells could not be attributed any distinct pattern. Altogether from the spatial analysis of the images and clustering analysis based on independent methods, the results strongly support the conclusions for a distinct difference between Act D and CHX treated cells and the proposal that transcription is recognised in the host cell and results in distinct events we have termed genome congregation (see discussion).

### HSV genome transport is independent of proteasome and nuclear export activity

**Fig 11:**
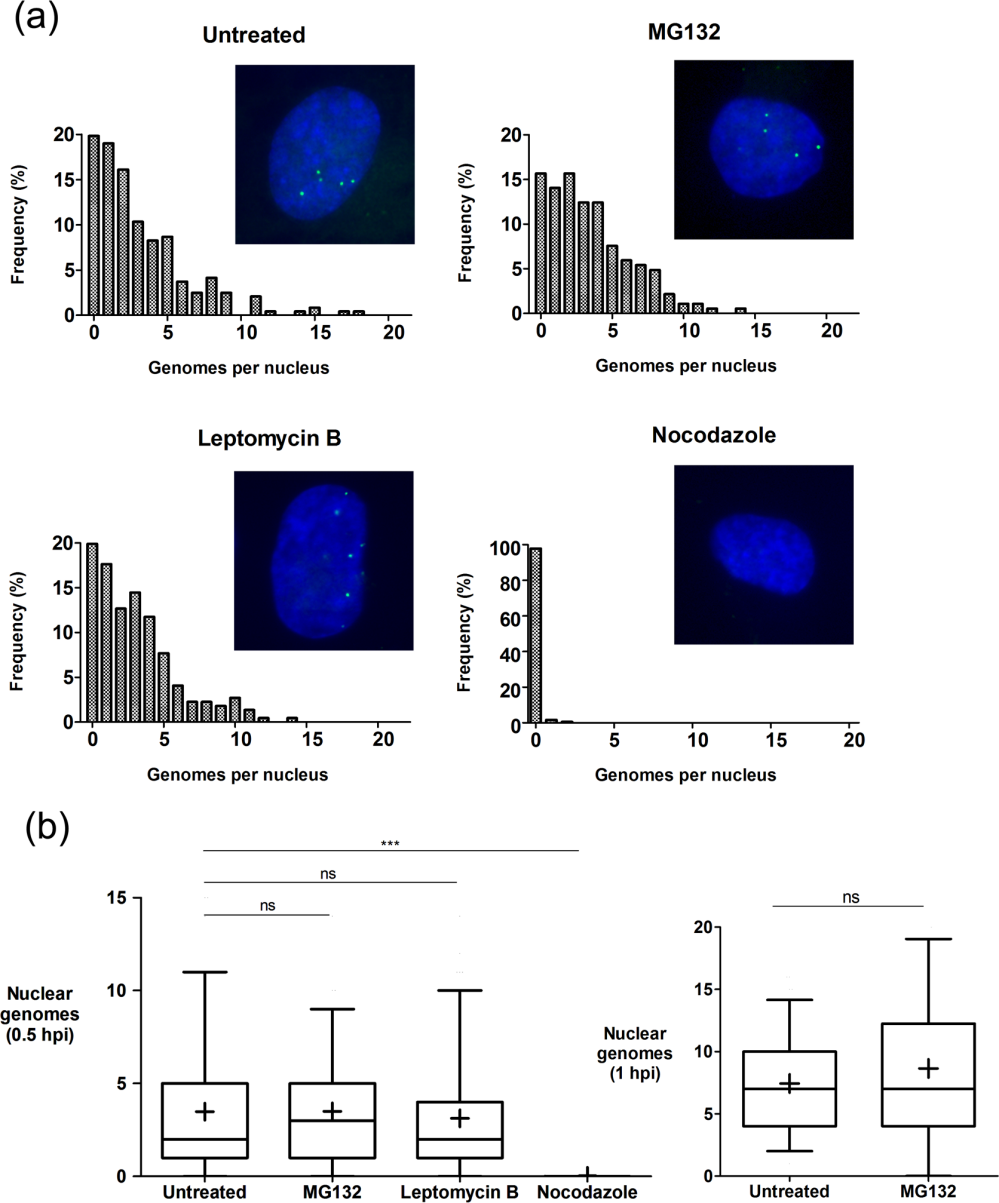
Effects of drug treatment on genome nuclear entry. Cells were mock-treated or treated with MG132 (10 μM), Leptomycin B (20 nM) or nocodazole (2 μM) as indicated. Inhibitors were added to cells for 1 hr prior to infection with HSV^EdC^ (moi 10). Cells were analysed at 0.5 hpi for the localisation of EdC-labeled genomes as described for other figures. For MG132 we also analysed genome localisation at 1 hr. (a) Each panels shows a representative image at high magnification (x63 objective) together with histograms of quantitative evaluation of the frequency of numbers of genomes/nucleus observed for each condition (at least 200 nuclei for each). (b) Box and whisker plots for data in (a). Box shows 2^nd^ and 3^rd^ quartiles with a horizontal bar in the middle showing the median, while whiskers show up to 5–95% of the total population. ‘+’ denotes the mean value. Unpaired two-tailed t-tests were used for statistical results (ns = not statistically significant, *** = p<0.0001). In this experiment infection even in the untreated sample was somewhat less efficient than standard, but there was no significant difference with either MG132 or Leptomycin B at 30 min and no diference for MG132 at 1 hr. In contrast, Nocodazole treatment resulted in a substantial and significant reduction in accumulation of uncoated nuclear genomes as discussed in the text.

In a previous report using an indirect surrogate measure (i.e., β-galactosidase enzyme activity from a recombinant virus or capsid localisation), it was concluded that proteasome inhibition, which suppressed β-galactosidase activity, did so by preventing HSV genome transport to the nucleus [37]. Having established a direct assay, we addressed whether proteasome inhibition had any detectable effect for genome uncoating and nuclear import. MG132 (10 μM) was added to cells 1 hr prior to infection with HSV^EdC^ at moi 10, and genome localisation assessed at 0.5 hpi compared to untreated cells. The distribution of numbers of nuclear genome foci in individual cells was assessed for at least 50 cells in each condition (Fig 11A, insets show representative individual nuclei). Overall there was no significant effect of MG132 on either total numbers or distribution of HSV genomes transported to the nucleus. Similar results were obtained analysing genome localisation at 1 hr (summarised, Fig 11B). Inhibition of CRM1-dependent nuclear export by Leptomycin B treatment has previously been shown to inhibit Adv genome nuclear entry [16]. We also examined the effect of Leptomycin B (as used in the previous studies). In contrast to the effects on Adv, we observed no significant effect on HSV^EdC^ genome uncoating and import. By comparison, when we examined nocodazole treatment, a drug which depolymerises microtubules and has been previously shown in many studies to inhibit HSV infection and capsid transport [38–40], we observed a very striking inhibition of the appearance of nuclear genomes. Control experiments for the activities of MG132 and Leptomycin B using known targets and effects confirmed their action (S8B Fig). Taken together with the positive control for the suppression of genome nuclear entry by nocodazole, we conclude that neither proteasome function nor nuclear export are required for the initial stages of HSV nuclear transport, uncoating and nuclear import.

## Discussion

**Fig 12:**
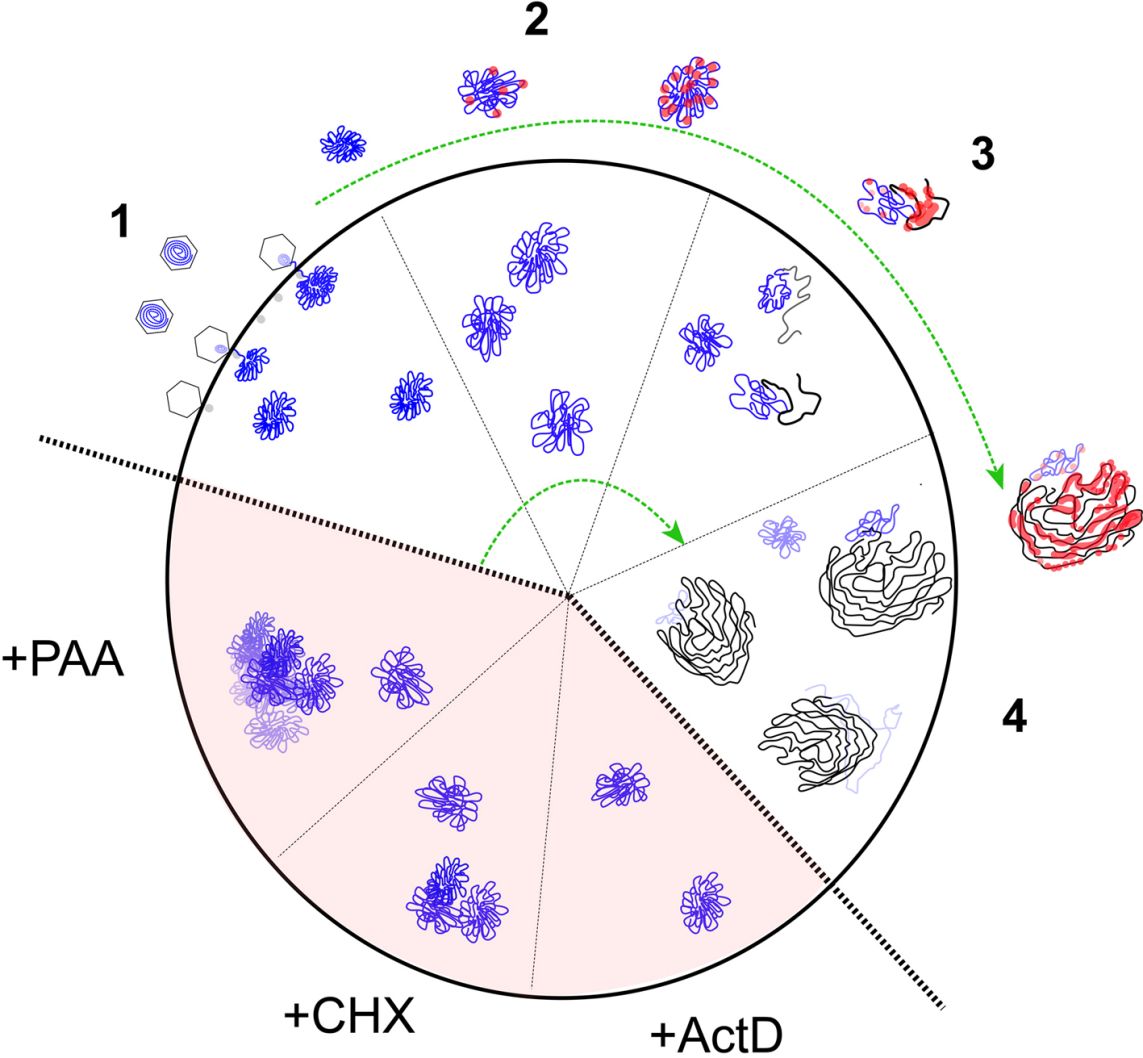
Model for HSV genome dynamics in nuclear entry, compaction and ICP4 association. We propose a model for spatiotemporal dynamics of the infecting HSV genome. The genome is indicated in blue. Progressive phases reflecting observations on certain qualitative features of genome organisation (which will naturally not occur completely synchronously), are demarked as phase 1–4. For clarity and ease of discussion, the inner part of the circle indicates only genomes, while the outer part indicates the association of genomes with the regulatory protein ICP4 (indicated in red). The bottom sections in shaded background indicate features delineated in the presence of inhibitors. Replicated progeny genomes are indicated in phases 3–4 in black. Details of the model are as discussed in the text.

Previous work has helped elucidate many aspects of the key processes of capsid transport within the cytoplasm, engagement with the nuclear pore, uncoating and genome transport into the nucleus [1, 4–9, 41–44]. Moreover for some viruses including HSV, the nature of the infecting genome has been extensively pursued by biochemical analyses, e.g., micrococcal nuclease (MCN) digestion [45–51] or by chromatin immunoprecipitation (ChiP) with antibodies to specific host cell histones or histone isoforms [52–55], Nevertheless altogether these studies give an incomplete understanding of infecting genome dynamics, at times difficult to reconcile [56] and with little insight into spatial aspects of transport, uncoating and organisation of the genome itself during the progressive stages or early infection. Thus many fundamental aspects of early genome dynamics remain poorly understood. In this work we focus on spatial aspects of genome dynamics during HSV infection with results which yield new insight into intracellular transport and organisation of the genome and help complement biochemical analyses to inform or qualify their interpretation. A summary of key conclusions and their implications from different aspects of this work is illustrated in Fig 12 and discussed below.

### Genome detection within HSV^EdC^ virions

We demonstrate the efficient incorporation of EdC into virus replication compartments, colocalisation with the major virus DNA binding protein, ICP8 and features of virus DNA replication in relation to the cell cycle that are entirely consistent with previous work [28–30]. However, results demonstrating the incorporation of EdC into replication compartments and its minimal effect on virus yields do not necessarily mean that it would be incorporated into mature infectious virions. Information on the efficiency and proportion of particles that contain detectable EdC is necessary for subsequent analyses and we exploited an in vitro assay described by Newcomb et al., who showed that HSV genomes are ejected from the capsid upon attachment to solid supports due to undefined structural perturbation(s) [26, 27]. Consistent with this, we show that adsorption on glass induces a structural change in the capsid permitting access to the catalytic molecules involved in cycloaddition, including the azide-fluorochrome. In our analysis with virions the genome was retained within the particle while with purified capsids the genome was readily released [26]. We propose that while there is some structural change in the virion capsid allowing the coupling reaction to the DNA, the genome is nevertheless retained in the confines of the particle due to surrounding components. It was also previously demonstrated that when the genome was released from purified capsids, it was ejected in a polarised manner likely from the portal and that the proposed structural alteration may impact directly or indirectly on portal integrity [26]. A portal-specific alteration is possible but not necessary to explain our observations and it could be that some more global perturbation around the capsid shell could allow access to the components of the cycloaddition reaction, the largest being the azide-fluorochrome (mol wt 861). Indeed there could be distinct perturbations with one type allowing access to small compounds and another involving changes including at the portal, promoting genome release. Whether this is the explanation for our observations is beyond the scope of this work. Nevertheless the ability to identify the genome within the capsid might be exploited for other types of analysis e.g. in vitro biophysical analysis of genome transitions [57] or the identification of specific host components that may promote release. It is also interesting to compare these results with similar analyses of Adv on solid supports [16]. In the case of Adv, EdC-labelled genomes were not detected upon initial adsorption of the virus to coverslips but were observed after heat disruption. Heat treatment revealed internal protein VII (by immunofluorescence) and allowed cycloaddition labelling of the genome, which nevertheless remained tightly associated with the capsid [16]. The ejection of the HSV genome (from capsids or heated virions) versus the maintained association of the Adv genome (from heated capsids) most likely reflects differences in the pressurisation status of genomes within capsids [58] and the lack of DNA packaging proteins within HSV compared to Adv where the genome is associated with several core proteins, in particular protein VII [59]. Such differences in internal pressure and protein-genome association are likely reflected in differences in mechanism in nuclear pore engagement and genome import.

### HSV genome entry

Uncoated nuclear genomes could be detected within 30 min of infection at 37°C with HSV^EdC^. Using similar methods to examine Adv infection, genomes could not be detected in the nucleus at 30 min and quantitative analysis on nuclear entry was performed at 2.5 hrs, a comparatively late point in our analyses, when genomes were already uncoated, decondensed and in many cases beginning to replicate. This does not necessarily indicate that HSV genome import is more rapid than adenovirus. This would require a direct parallel comparison with similarly labelled viruses in the same cells and on identical imaging systems and even then would be a qualified comparison. Nonetheless for the majority of Adv capsids, their genomes become rapidly accessible to click detection in the cytoplasm, reflecting the initial stages of uncoating [12, 16, 59, 60]. Given differences in entry processes it is perhaps not unsurprising that unlike Adv, HSV genome entry is not sensitive to Leptomycin B inhibition of nuclear export. For HSV^EdC^ while uncoated cytoplasmic genomes could be detected this was a minor population of the total genomes, mostly in a subpopulation of cells. Although unlikely to contribute to the nuclear genome pool, such genomes may play a distinct role in the cell population as a whole, including host responses from subsets of cells that might elicit paracrine effectors to other cells. We conclude that for HSV, most capsids do not undergo structural transitions that perturb access (at least resolvable by cycloaddition labelling with azide-fluorochromes), such transitions likely being tightly coupled to engagement with the nuclear pore. However other factors including cell type could influence capsid integrity and genome accessibility, e.g., differences in entry by fusion at the plasma membrane versus endocytosis. The ability to positively identify genomes associated with perturbed capsids will be useful in future studies including investigation of capsid integrity as a function of cell type and the influence of prior immune stimulation by different pathways.

We found no significant qualitative or quantitative difference in the presence of MG132 and conclude that proteasome function is not required for transport of the relevant capsid population or genome import per se. This is in contrast to a previous publication which indicated that proteasome function was required post-entry for efficient delivery of incoming HSV capsids to the nucleus and subsequent gene expression [37]. While there could be several explanations for the difference, in our analyses we directly measure genome import while previous conclusions were based on measurements of gene expression (β-galactosidase) at 6 hpi or on measure of capsid localisation of a fluorescent virus at 2.5 hpi. Proteasome inhibition could have inhibited a number of processes involved in surrogate read-out later in infection or inhibited bulk capsid dynamics that were not important for early genome delivery.

As summarised (Fig 12), at the earliest time detectable (stage 1, within 0.5 hpi) uncoated nuclear genomes were in a comparatively homogeneous, roughly spherical form that had expanded to approximately 3-fold the volume within virions. Thus while there is a distinct genome decompaction after nuclear import, this is constrained in a relatively regular manner. The distribution of the numbers of genomes appearing in the nucleus and the relationship to moi bear similarity to those from physical analysis of adenovirus genome entry [16] and are relevant also to conclusions from other studies on the numbers of herpesvirus nuclear genomes that participate in transcription and replication [61–63]. We observed that at a standard moi of 10 pfu/cell (100–200 particles coating a cell), although a small percentage of nuclei could contain relatively high numbers, the mean numbers of nuclear genome foci was approximately 5 and around 90% of cells had fewer than 10. Increasing moi by 5-fold did not increase nuclear genome numbers 5-fold indicating the operation of some form of limit(s) on infection (though this could be at a number of stages) and a decreasing efficiency of nuclear import at higher mois. Similar conclusions were made for Adv nuclear entry [16]. We currently cannot discriminate the fate of capsids which do not uncoat at the nuclear pore, while for Adv genomes appear to be lost from the capsid. Based on mathematical modelling of simultaneous infections with strains of pseudorabies virus expressing individual fluorophores, it has also been estimated that an average of approximately five infecting genomes are expressed per cell at a moi of 10 and that even at moi 100 the mean is no more than 7–8 genomes [61]. Clearly, at the more extreme ends of distributions from our analyses, high numbers of genomes can enter the nucleus. However, at a standard moi of 10, the mean numbers of physical nuclear genomes are of the same approximation as the numbers of genomes that have been proposed to express or replicate [61]. One implication from this is that once imported into the nucleus, the efficiency of transcription may be relatively high. Indeed at early times, many of the physical genomes were associated with ICP4 (see below), though this does not necessarily mean such genomes are indeed transcribed. Such conclusions will require the ability to simultaneously visualise genomes and nascent transcripts or accumulating RNA. We are currently developing bioorthogonal approaches [64] with distinct chemical moieties on DNA versus RNA that allow copper-dependent and independent coupling of distinct fluorochromes to examine these questions in virus infected cells.

### Genome compaction state transitions during infection

After nuclear import, the HSV genome initially expands and continues to decondense in a series of transitions that could be delimited using chemical inhibitors. In the absence of transcription, genomes remained relatively compact and were maintained in that form in the nucleus for at least 8 hrs. De novo virus protein synthesis is therefore not required for the initial compact state of the genome, the nature of which is discussed further below. Allowing transcription but in the absence of de novo translation revealed a distinct feature, which we termed genome congregation, not observed when transcription was blocked. Several mechanisms could contribute to this process. Transcription on the viral genomes themselves, i.e., the templates for transcription, could directly contribute to congregation, even if not all genomes were transcribing. This could be via components of the transcription/splicing apparatus somehow progressively capturing multiple genomes in a spatially restricted manner. It could also be that genome congregation is a host response to infection (with no viral proteins yet made), specifically recognising the process of transcription and sequestering genomes as a result. Further explanations are possible but understanding the process of genome congregation will be important for any full understanding of genome dynamics, competency for transcription and host responses to infection (see below).

During normal infection, genomes underwent further progressive decondensation, eventually becoming difficult to discriminate and dissipating within DNA replication compartments. We frequently observed longer lived residual condensed foci usually at the periphery of replication compartments (Fig 12, stages 2–4). The intermediate stages of these genome transitions did not require DNA replication per se, with the enlarged and decondensed morphology of infecting genomes in the presence of DNA replication inhibitors being distinct from that observed in the presence of Act D or CHX (Fig 12, summary schematic view, shaded sectors). We conclude that recruitment of regulatory and/or replication factors combined with the more extensive early transcription, results in further changes and decondensation of the genome while downstream DNA replication and associated processes e.g., recombination, branching and extensive late transcription, [65, 66] results in more complete decondensation of input genomes at later times. It is possible that the longer lived condensed foci remaining on the periphery of replication compartments represent either replicated parental strands that remain as foci, or potentially a subset of parental genomes that were not acted upon by either replication or transcription. Rolling circle replication [65, 67, 68] acting on HSV^EdC^ would result in one labelled parental strand remaining at the replication fork, while the other parental strand would progressively move away as replication and unlabelled progeny DNA accumulates. It is not currently technically possible to discriminate between these possibilities and other explanations are also possible but in this regard the pattern of genome association with ICP4 warrants discussion.

### ICP4 association with infecting HSV genomes

While there was heterogeneity at an individual genome level with some genomes not accumulating ICP4, the majority of condensed genomes recruited and were enriched for ICP4 by 1–2 hrs. (As discussed above, this does not necessarily imply productive transcription from all genomes). However as infection progressed there was a clear distinction in this association. Those genomes that remained as condensed foci (found mainly on the periphery of replication compartments) were selectively depleted for ICP4 and frequently devoid of the protein altogether. One possible explanation is that these foci never accumulated ICP4, in which case they exhibit significant selectivity since ICP4 would clearly have been initially recruited to certain other foci and ICP4 was also present in adjacent decondensed replication centres in the same nuclei. Alternatively it could have been that ICP4 was recruited to many foci but different downstream pathways dictate either maintenance of a more condensed state coupled with displacement of ICP4 or progressive decondensation (and associated replication/transcription) and association with ICP4. Future work developing methods for the simultaneous visualisation of infecting genome localisation and condensation, active transcription and protein localisation will help address the nature of these relationships revealed in this work.

Finally, our results on spatial analyses are relevant to the interpretation of the many previous biochemical analyses on the nature of the infecting HSV genome. MCN digestion experiments of the bulk virus genome population strongly indicate that the considerable majority of infecting genomes released from the capsid are randomly digested and not assembled into any conventional nucleosomal organisation [45–50, 69]. On the other hand ChiP analyses, which usually address a minor fraction of the total DNA, suggests that histones in some form are associated with at least a population of genomes [52, 53, 55, 70, 71]. One model attempting to integrate results from different approaches proposes that infecting genomes associate with some form of nucleoprotein complex that includes histones but in a non-conventional highly distributive, rapidly associating/dissociating organisation [56, 69]. We show that after capsid exit and nuclear import, genomes expand but in a constrained and distinct state and then further decondenses in a discernible fashion prior to replication, and that replication and potentially associated transcription result in further extensive dissipation within the nucleus. In addition to heterogeneity arising from overlapping temporal transitions, heterogeneity arises from subpopulations of genomes that may not associate with e.g. ICP4, or which at later times remain in a more condensed configuration. Thus certain proteins may be selectively associated with specific subpopulations of these genome, as an example the longer lived condensed foci, and thus antibodies to such proteins sample only those genome populations. Future work combining bioorthogonal chemistry for spatial analyses of genomes and ongoing transcription and replication together with immunofluorescence analysis to localise viral and host cell proteins will be necessary to resolve these questions.

In conclusion, using compatible bioorthogonal nucleoside precursors for genome labelling in HSV infected cells and quantitative individual particle analysis, we demonstrate extremely efficient precursor incorporation resulting in virtually quantitative detection on an individual particle basis in the population of progeny virus. We then report a comprehensive analysis in infected cells of genome dynamics during capsid exit and nuclear import in which we; demonstrate qualitative transitions in genome condensation state linked to transcription and replication; reveal novel processes in genome congregation dependent upon transcription and show the temporal switching in regulatory protein recruitment (represented by ICP4) to distinct genome compartments. Altogether our results reveal novel aspects of the spatiotemporal dynamics of HSV genome uncoating, transport and organisation that can be integrated with previous biochemically based analyses and provide a framework for future investigation in distinct fields of host cell-virus genome.

## Materials and methods

### Cell culture, viruses and infections

RPE-1 cells, a human telomerase immortalised retinal pigment epithelial cell line, (kindly provided by Dr Andrew McAinsh University of Warwick, UK) were grown in Dulbecco’s modified minimal essential medium (DMEM/F12, Sigma-Aldrich) supplemented with 200 mM glutamine, 10% newborn bovine serum (NCS; Gibco) and penicillin/streptomycin. The wild-type (w/t) parental strain was HSV-1[17]. Routine plaque assays were performed in RPE cells in the presence of pooled neutralising human serum (Sigma-Aldrich) at 2% or clinical grade purified human immunoglobulin (IVIg, Carimune NF, Nanofiltered, human immune globulin, CSL Behring) at 2 mg/ml, having demonstrated complete neutralisation of extracellular virus at this dose (>6 log reduction in virus titre). High multiplicity infections were performed at multiplicities of infection stated in the experiments and for routine experiments usually at a moi of 10. In control experiments for genome uncoating, the inoculum was treated with 500 U/ml DNase I (Roche) for 1 hr at 4°C, or 10 mg/ml IVIg for 0.5 hr at room temperature. Inhibitors were used at the following final concentrations; acycloguanosine (ACV, Thermo Scientific, 500 μM); phosphonoacetic acid (PAA, 400 μg/ml); actinomycin D (Sigma-Aldrich, 5 μg/ml); MG132 (Calbiochem, 10 μM); Leptomycin B (Sigma-Aldrich, 20 nM); nocodazole (Sigma-Aldrich, 2 μM). Inhibitors were added to cells for 1 hr prior to infection.

### Assessment of EdC labelling on cell viability and virus yield

To examine the effects of EdC on cell growth, RPE-1 cells were pulsed with increasing concentrations of EdC (Sigma-Aldrich, #T511307) for 48 hr and examined by phase-contrast microscopy either live or after fixation and staining with crystal violet. Viability was determined by trypan blue exclusion using an automated cell counter. For the examination of the effects of EdC pulse-labelling on viral plaque development, RPE cells were infected at 50 pfu/well, the inoculum was then neutralised with 2% human serum after 1 hpi, and EdC was then added 2 hpi for the remainder of the assay. Plaque sizes and numbers were measured at 48 hpi using Image Pro Plus 7 software. For the effects of EdC on virus yield, RPE-1 cells were infected at moi 5 for single-step growth or moi 0.005 for multi-step growth. Inocula were neutralised at 1 hpi with a 40 mM citric acid wash. Cells were then incubated in the presence of various concentrations of EdC (added at 2 hpi) for the duration of the experiment. Supernatant and cell-associated virus was harvested at 20 hpi (single-step) and 72 hpi (multi-step) and yield assessed by plaque titration on RPE-1 cells.

### Production of HSV-1 containing EdC-labelled genomes (HSV 1EdC)

RPE-1 cells were grown in roller bottles (850 cm^2^ surface area) to ~80% confluency. The cells were infected with HSV-1[17] at a moi of 0.025 in 25 ml medium without serum and made to 2% NCS at 1 hpi. EdC was added to a final concentration of 5 μM at 6 hpi and again at 24 hpi. Virus was harvested at approximately 48 hpi, separating cell associated and supernatant virus by low speed centrifugation (3000 rpm, 4°C for 15 min). Supernatant virus was transferred into Oakridge tubes and pelleted in a Sorvall centrifuge RC5B using a SS34 rotor at 19,000 rpm at 4°C for 90 min. For cell-associated virus, the virus pellet was first clarified of cell debris, pelleted by high speed centrifugation as above, resuspended in PBS and applied to the top of 0.5 ml 35% sucrose cushion in polyallomer tubes and centrifuged at 25,000 rpm in a SW55Ti rotor for 1 hr. The virus pellet was resuspended and stored in PBS. Virus titres were determined on RPE-1 cells. Particle/pfu ratios were calculated by diluting control stocks of HSV or HSV^EdC^ to equal pfu titres, spotting standardised aliquots onto coverslips and enumerating total VP5 capsid-containing virions by automated immunofluorescence microscopy and multi-tiled image acquisition to capture and quantify the entire population. Total VP5-positive/pfu particle numbers could then be evaluated for each stock. Alternatively we examined protein profiles of standardised amounts of purified virus and quantified the amounts of the major capsid protein as previously described [72]. Similar results were obtained in comparing HSV and HSV^EdC^ particle/pfu ratios by the two methods. The ‘Mock EdC’ inoculum used for control experiments was prepared by pulsing uninfected RPE-1 cells for 48 hr with 5 μM EdC, harvesting the supernatant and concentrating exactly as if preparing virus from infected cells.

### Cycloaddition of labelled fluorochromes and immunofluorescence

Cells grown on borosilicate coverslips, infected and labelled with EdC under the variety of experimental conditions discussed, were fixed in 4% paraformaldehyde (PFA) in phosphate-buffered saline (PBS) for 10 min, quenched in 100 mM glycine in PBS for 5 min, and permeabilised with 0.5% Triton X-100 for 5 min. Samples were then processed by cycloaddition with azide-linked fluorochromes and then blocked with 10% FBS in PBS where immunofluorescence was required. Cycloaddition and immunofluorescence were essentially as described previously [24]. Briefly for the cycloaddition reaction to detect EdC labelled DNA, PFA fixed and washed coverslips were incubated in freshly prepared reaction buffer containing 1 mM CuSO_4_; 10 mM sodium ascorbate; 10 mM amino-guanidine and 1 mM Tris(3-hydroxypropyltriazolylmethyl)-amine (THPTA, Sigma-Aldrich) and 10 μM Alexa 488-azide (Thermofisher) in PBS pH 7.4. Reactions were performed for 2 hr in the dark, the reaction cocktail then removed and the samples washed with PBS, dried and mounted. For subsequent immunofluorescence, cells were blocked and stained for 45 min with primary antibodies and 45 min with secondary antibodies by standard methods and mounted in ProLong Gold Antifade Mountant (Molecular Probes). Images were acquired with Zeiss Axiovert 135 TV microscope using Zeiss x63 lens (Plan-APOCHROMAT, 1.4 numerical aperture) and Retiga 2000R camera with Image Pro Plus 7.0 software.

### Antibodies for immunofluorescence studies

The following antibodies were used: mouse anti-VP5 (Virusys, HA018; 1:300); mouse anti-ICP8 11E2 (Abcam, #20194; 1:100); mouse anti-ICP4 (Virusys, H1A021; 1:400); mouse anti-α-tubulin (Sigma-Aldrich, #T6074; 1:1000); rabbit anti-PML [73] (1:300); mouse anti-cyclin B1 (Abcam, ab18221; 1:500); Alexa-594 Goat anti-mouse IgG (Thermofisher; 1:750).

### HSV^EdC^ genome detection in virions

Samples of HSV-1[17] wild-type or HSV^EdC^ at 1x10^8^ pfu/ml were applied to borosilicate coverslips, adsorbed for 15 min, fixed with PFA and processed as above to detect genomes and immunofluorescence using anti-VP5 antibody to detect capsids. For experiments examining genome exit we used procedures as previously reported [26, 27] where virions absorbed onto the coverslips were subject to heat treatment (70°C for 2 min) either before or after PFA fixation. Samples were then processed for detection of the genome and capsids as before. In parallel experiments, viruses were subject to the cycloaddition reaction in PBS containing the appropriate concentrations of reagents. After the reaction, samples were made to 1 mM EDTA to stop any further reaction, then adsorbed onto coverslips and processed for immunofluorescence. For quantitative analysis maximum projections were captured using the Image Pro Plus Stage-Pro function and Z-stacks were obtained with 10 slices at 0.2 μm intervals. We used Image J and a customised plugin based on the find maxima protocol. The plugin uses find maxima and places an identical sized ROI centred on the maxima with user configurable diameter to encompass virus particles. Maxima with too close a spatial overlap or at an image edge are excluded by the protocol and can be further excluded manually before quantitation. In practice this had a limited effect given the largely monodisperse nature of analysed particles. Red (capsid) and green (DNA) intensities were measured for each ROI. Mean and standard deviation (SD) background intensities were calculated separately for the red and green channels from the area outside the identified ROIs and normalised for ROI area. Maxima–based ROIs were then compared separately against the mean background for each channel and categorised using a threshold the default of which was the mean channel background plus 1 SD. Thus, to be categorised as a red (capsid) positive particle, that particle ROI must be not only be above the mean background ROI in the red channel but at least 1 SD above that background. Frequency distributions of individual identified particle ROIs were then quantitated, calculating the bin width using the Friedman-Diaconis criteria for interquartile-ranges [74, 75]. The same bin width was used for both channels in the figure for ease of comparison of the distributions. Gaussian distributions were fitted to each channel frequency data using Image J curve fitter.

### Analysis of intracellular genome uncoating

Cells grown on coverslips were mock-infected or infected with parental HSV-1[17] or HSV^EdC^ by normal procedures (moi 10) at 4°C for 45 min to allow virus adsorption to the cell surface. Cultures were then either washed and fixed immediately for analysis of adsorbed virus or shifted to 37°C to allow the infection to proceed. For analysis of infected cells at very early times i.e., 30 mins, the inoculum was then removed and cells washed and fixed. For longer times, the inoculum was removed and replaced with pre-warmed medium containing 2% NCS. Cultures were washed and fixed at various times thereafter as indicated in the text and figure legends and processed for genome detection by cycloaddition reaction and immunofluorescence as described above. Infected cells were co-stained with DAPI to allow outlining of nuclei. Images were acquired by standard wide-field microscopy (described above) or 3D-structured illumination microscopy (3D-SIM). Images were then processed using Image J denoise plugin and corrected for background. For quantitative evaluation of genome foci, images were imported into Image Pro Plus and then subject to thresholding and segmentation modules to define object masks which were quantified for various parameters. Using the DAPI outlines and the population analysis tool, the number of genomes within approximately 200 nuclei was calculated for each condition under study, differing mois, times and various drug treatments.

### 3D-SIM

Super-resolution imaging was performed on Elyra PS1 system (Carl Zeiss) with an Apochromat 63x 1.4 NA oil objective lens, 488nm and 561nm excitation lasers and images were captured on a sCMOS PCO Edge camera. The camera pixel size is 6.5 μm and with 63x objective and additional 1.6x tube lens, this corresponds to 64 nm in the object plane. For analysis of infected cells, image stacks (2 μm) were acquired in Frame Fast mode (single multiband cube) with a z-step of 110 nm and 15 raw images (five phases, three angles) per plane. Raw data was then computationally reconstructed using the ZEN software to obtain a super-resolution 3D image stack with a pixel size of 32 nm in xy and 105 nm in z. The SIMCheck ImageJ/Fiji plugin [76] was used to perform quality control on both raw and reconstructed data and to estimate lateral (x-y) resolution (approximately 120 nm) and axial (z) resolution (approximately 300 nm). Images from the different colour channels were registered in ZEN with alignment parameters obtained from calibration measurements with either virus capsids simultaneously labelled in both red and green channels or with TetraSpeck calibration beads 0.1 μm diameter (Thermofisher). 2D Gaussian fitting was done using the PALM analysis function in Zen with 30 pixel image window or ‘GaussFit on spot’ plugin in ImageJ. The Gaussian 1/ sqrt (e) radii were converted to full width at half maximum (FWHM) values by multiplying with 2x sqrt (2*ln(2)).

For analysis of HSV^EdC^ capsid and genome dimensions by immunofluorescence and cycloaddition reactions, the expected dimensions can be estimated by a convolution of the SIM resolution (120 nm and 138 nm for 488 nm and 561 nm excitations respectively) with the known sizes of capsid diameter (125 nm) and genome space (100 nm) [77]. In case of the capsid by immunofluorescence, based on previous analyses [78] we estimated and additional 35 nm for the primary/secondary antibody bringing the estimated dimensions of the capsid to 160 nm. The convolutions of the SIM resolution and these sizes results in an estimated size of 200 nm and 170 nm for the capsid and genome respectively. Our measured average size is about 26% smaller than the estimate size. This was in line with measurements of calibration measurements with standard fluorescent beads of different sizes where the FWHM sizes were found to be 22% smaller than expected.

Volume analysis was performed the object analyser module of the Huygens image processing suite (SVI, The Netherlands). The image is segmented into defined objects by the seed-threshold level adjustment, and connection process. Introduction of a watershed increases segmentation reliability further. Detected objects are automatically labelled and submitted to a continuous Iso Surface renderer. The segmented image is shown as a coloured iso-surface image. Object statistics are reported for each object, including geometrical data and spatial location. A simulated fluorescence process (SFP) computing algorithm allows visualization of the 3D data and production of the rendered image as an animation. Using this method we estimated volume and sphericity for genomic foci from visions on coverslips versus after entry to the nucleus.

### Spatial analysis and clustering

Spatial clustering analysis of EdC labelled genomes was carried out using the Spatial Statistics 2D/3D ImageJ plugin [35]. The plugin analyses the overall distribution of inter-point distances including any local clusters and calculates whether there is evidence for a non-random distribution in the population of cells. A binary mask of each nucleus is generated together with a mask of the genomes using the ‘Find Maxima’ function. The plugin calculates for every nucleus, the distances between every point and its nearest neighbour and generates a cumulative distribution function (CDF) of those distances (the G-function). To compute this function, first the average CDF of a completely random distribution is estimated over a set (500 iterations) of randomly generated point patterns, specific to each reference structure (nucleus) and the number of points (genomes) in that structure. Second, the expected variation of CDFs around their average is estimated using a second set of randomly generated binomial point patterns. The relative position of the observed CDF for the actual test set within this range of variation is used to assign a p-value to the observed pattern, termed the ‘Spatial Distribution Index’ (SDI). Point patterns that tend to clustering have an SDI closer to 0 while patterns tending to even spacing have an SDI close to 1. The CDFs of SDIs of two different populations are compared using the Kolmogorov-Smirnoff (KS) test, which is non-parametric and distribution free. A p-value for the difference between the two populations is calculated, as well as the D statistic which is the largest deviation between the two CDFs.

An independent clustering analysis was performed by calculating the Ripley function (K) using the BioImage Analysis platform ICY (http://icy.bioimageanalysis.org) as described [79]. Nuclei were segmented using the DAPI signal as above and a binary mask created. The ICY Spot Detector plugin identifies the EdC genomes contained within the nuclear mask. These were used to calculate the K function using the Spatial Analysis plugin [36]. In this approach regions of interest (circles) with increasing radii are drawn around every detected spot and other spots located within the circles are identified in the overall search area (the nucleus). The K function is then based on the number of spots that are closer than the radius, calculated for each increasing radius. The function is used to report the statistical significance of whether a distribution of points is random or clustered by comparing obtained values with critical quantiles under a completely random distribution. The amplitude of the K-function can then be compared to corresponding low and high quantiles (0.01 and 0.99 here). When K is higher than the high quantile for a certain radius, the foci are significantly organised in clusters. Conversely, when K is lower than the low quantile, the foci are dispersed.

## Supporting information

S1 Fig EdC has no effect on RPE-1 cell growth. (a) RPE-1 cells were grown in the continued presence of EdC at various concentrations for 48 hr. Images illustrate normal numbers and morphology of live cells at the end of the incubation period (scale bar 100 μm). (b) RPE-1 cells (3 x 10^5^) were plated and grown in the presence of EdC at various concentrations for 48 hr. Viable cells were quantified by trypan blue exclusion at the end of the incubation period.
(TIF)

S2 Fig EdC incorporation during S-phase. RPE-1 cells were pulsed for 4 h with 5 μM EdC, and processed. Incorporated EdC was detected by cycloaddition using Alexa 488-azide capture reagent and cells counterstained with DAPI. Examples of different localisation patterns in uninfected S-phase cells (labelled i-iv as discussed in the text) are illustrated (scale bar 10 μm).
(TIF)

S3 Fig Simultaneous analysis of distributions of VP5 (capsid) and EdC (DNA) signals for individual particles. Quantitative analysis of individual particles was performed as described for Fig 4 and in materials and methods. We used a customised Image J plugin based on the find maxima protocol accompanied by pixel quantification in defined ROIs for each channel (top panel VP5, red; bottom panel EdC, green). Particles were scored positive by being not only above ROI normalised background threshold, but being at least 1 SD above that value. Frequency distributions of signal intensities for each channel of individual VP5 positive particles were quantitated with bin width automatically selected using the Friedman-Diaconis criteria for interquartile-ranges [74, 75]. The same bin width was used for both channels aligning means for both channels for ease of comparison of distributions. The raw mean and SD together with the coefficient of variance are reported on the right-hand side, with fitted means and SDs reported on the left hand side after Gaussian distributions were fitted to each channel frequency data using Image J curve fitter.
(TIF)

S4 Fig Scatter plot analysis of VP5 and genome signals for individual particles. The same analysis used for overall frequency distributions of VP5 and DNA of the total particles (S3 Fig) was used to generate scatter plots where each dot represents an individual particle and its score for VP5 (Y-axis) and EdC (x-axis). The threshold for scoring positive is indicated by the solid lines in red or green. Note that thresholds are calculated with regard to individual images and background for data in each field and may be marginally different. Particles for HSV^EdC^ that are positive for both signals are in the upper right quadrant and coded yellow. Particles which are VP5 positive and below threshold in green are coded red. For HSV w/t essentially no VP5 positive particle exhibited any significant green signal.
(TIF)

S5 Fig HSV^EdC^ genomes are only detectable after adsorbtion onto a solid support. (a) HSV-1^EdC^ virus particles were adsorbed onto borosilicate coverslips, detected by cycloaddition and immunofluorescence and analysed as described for Fig 4. (b) HSV-1^EdC^ was examined by cycloaddition in solution prior to adsorption on coverslips. The reaction was then blocked by addition of 1 mM EDTA to chelate the copper catalyst and the sample then adsorbed to borosilicate coverslips, stained for VP5 and analysed by ImageJ. Graphs show quantified data of particles under each condition for VP5 and EdC. (c) HSV-1^EdC^ was adsorbed to borosilicate coverslips as above and then heated to 70°C for 2 min before fixation and detection by cycloaddition and immunofluorescence for VP5. VP5, red; EdC, green (scale bar 10 μm).
(TIF)

S6 Fig Control experiments on entry and uncoating of HSV-1^EdC^ genomes. RPE-1 cells were infected with HSV-1^EdC^ and fixed at 2 hpi for detection by cycloaddition and immunofluorescence for VP5 (scale bar 10 μm). Experimental variations from the normal process were as follows: (i) During the cycloaddition, Cu(I) was omitted from the reaction mixture; (ii) the virus inoculum was treated with clinical grade neutralizing antibody IVIg (100 mg/ml) for 0.5 hr at room temperature prior to infection; (iii) the inoculum was treated with DNase I (500 U/ml) for 0.5 h at 10°C prior to infection; (iv) cells were infected with a ‘mock’ inoculum which consisted of concentrated supernatant from uninfected RPE-1 cells pulsed and prepared as for an infected HSV-1^EdC^ stock.
(TIF)

S7 Fig Video of iso-rendered 3D-SIM data of HSV^EdC^ infected cell. 3D-SIM data of a representative RPE-1 cell infected with HSV-1^EdC^ (moi 20) at 0.5 hpi, iso-rendered in Huygens analysis software as described in materials and methods. Channels have been rendered partially transparent for ease of inspection of features as discussed in the text Red channel, VP5; Green channel, EdC; blue channel, DAPI.
(AVI)

S8 Fig Control experiments on effects of drug treatments. (a) Representative images of the localisation of ICP4 in cells infected with HSV-1^EdC^ (moi 10) and either untreated or incubated in the presence of ActD (5 μg/ml), CHX (100 μg/ml), or ACV (500 μM), PAA (400 μg/ml), as indicated. Cells were fixed (3 hr post infection) and processed for immunofluorescence of ICP4. Scale bar 10 μm. (b) Uninfected cells were treated with MG132 (10 μM), nocodazole (2 μM), or Leptomycin B (20 nM) for 1.5 hr, as during analysis of infection, then fixed and processed for immunofluorescence for PML, α-tubulin, or cyclin B1 respectively (red channel). Cells were counterstained with DAPI. Panels I and IV show MG132 treatment increases PML number and size per nuclei; panels II and V demonstrate microtubule depolymerisation upon nocodazole treatment; panels III and VI show inhibition of nuclear export and nuclear accumulation of cyclin B1. Scale bar for MG132 and nocodazole panels, 10 μm; scale bar for Leptomycin treatment, 100 μm
(TIF)

S9 Fig Validation of spatial distribution index using a random sample. (a) Schematic illustration of theoretical random, clustered or evenly spaced patterns of foci in an individual cell. The spatial distribution analysis examines the overall distribution of inter-point distances, including any local clusters, and calculates whether there is any evidence for a non-random distribution in the population of cells. (b) The distances between every point and its nearest neighbour are generated as a cumulative distribution function (CDF) of those distances (see materials and methods). A theoretical CDF for a truly random distribution (black line; 95% confidence limits, grey lines) is generated (specific for each nucleus and number of foci) and then compared with the actual distribution obtained for the test set (red line). For an actual random pattern the red line will be close to the black, while for clustered and evenly spaced the red line will deviate left or right respectively. This difference is transformed to a spatial distribution index (SDI). Point patterns that tend to be clustered have a SDI closer to 0 while evenly spaced patterns have a SDI closer to 1. (c) The corresponding SDI frequency distributions between 0 and 1 for populations of cells. The CDFs of SDIs of different populations are compared using the Kolmogorov-Smirnoff (KS) test, which is non-parametric and distribution free. A p-value for the difference between the two populations is calculated, as well as the D statistic which is the largest deviation between the two CDFs. (e) Capsids applied on coverslips were fixed, stained with anti-VP5 and images processed to calculate the SDI, expected to be random. (f) The results compare the theoretical cumulative inter-point distances for a random distribution (black line; 95% confidence limits, grey lines) with the actual distribution obtained for the capsids (red line). The capsid distribution precisely conforms to a random distribution. Note while clusters can be observed, such clusters will occur randomly, by definition. The algorithm calculates whether there is any evidence overall for a significant non-random distribution.
(TIF)
