# Supplementary figures and images for: Spatiotemporal dynamics of HSV genome nuclear entry and compaction state transitions using bioorthogonal chemistry and super-resolution microscopy
Source: PLoS Pathog. 2017 Nov 9;13(11):e1006721. doi: 10.1371/journal.ppat.1006721 (PMC5697887; doi:10.1371/journal.ppat.1006721)

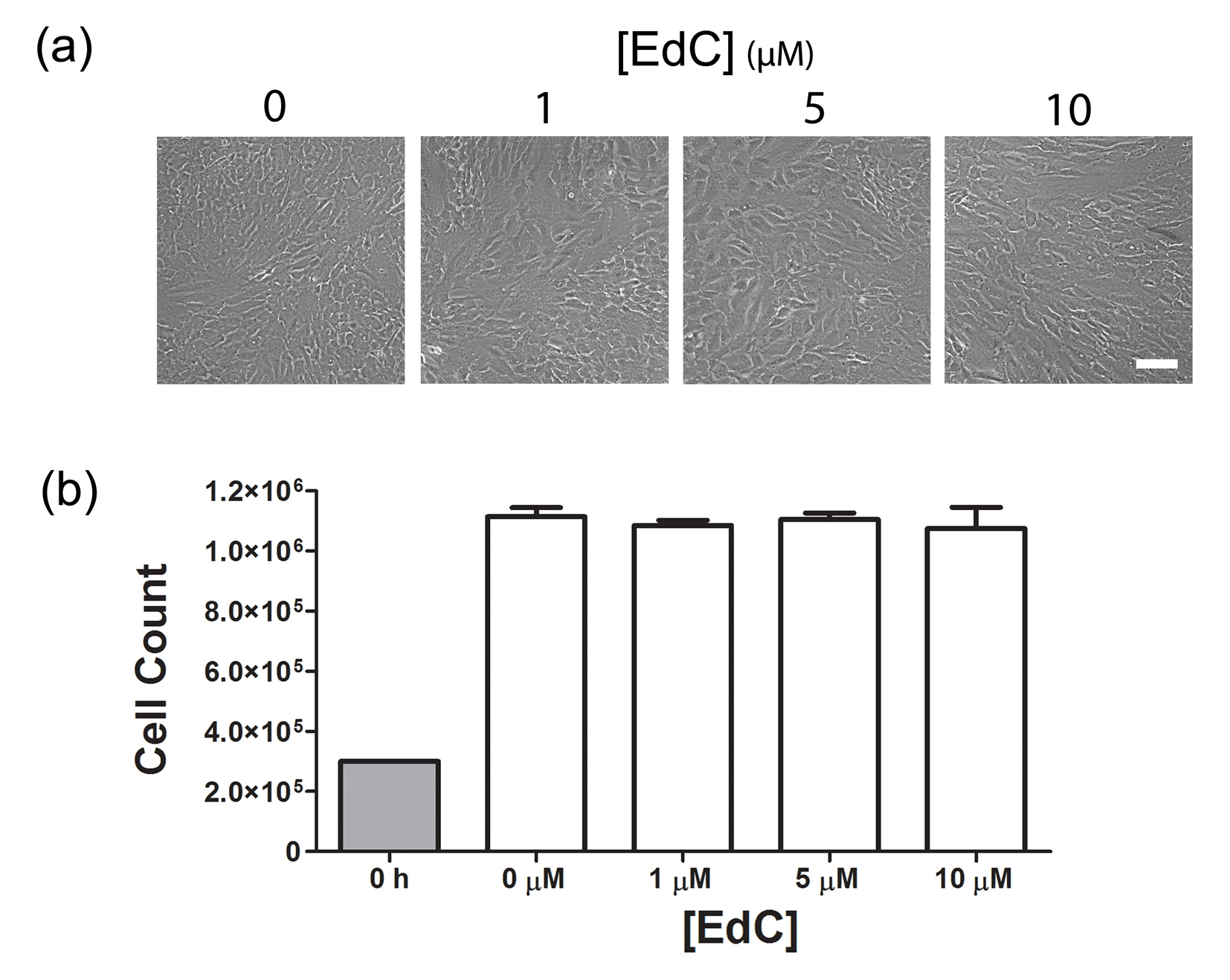

Supplement: S1 Fig — (a) RPE-1 cells were grown in the continued presence of EdC at various concentrations for 48 hr. Images illustrate normal numbers and morphology of live cells at the end of the incubation period (scale bar 100 μm). (b) RPE-1 cells (3 x 10^5^) were plated and grown in the presence of EdC at various concentrations for 48 hr. Viable cells were quantified by trypan blue exclusion at the end of the incubation period. (TIF) [file ppat.1006721.s001.tif]

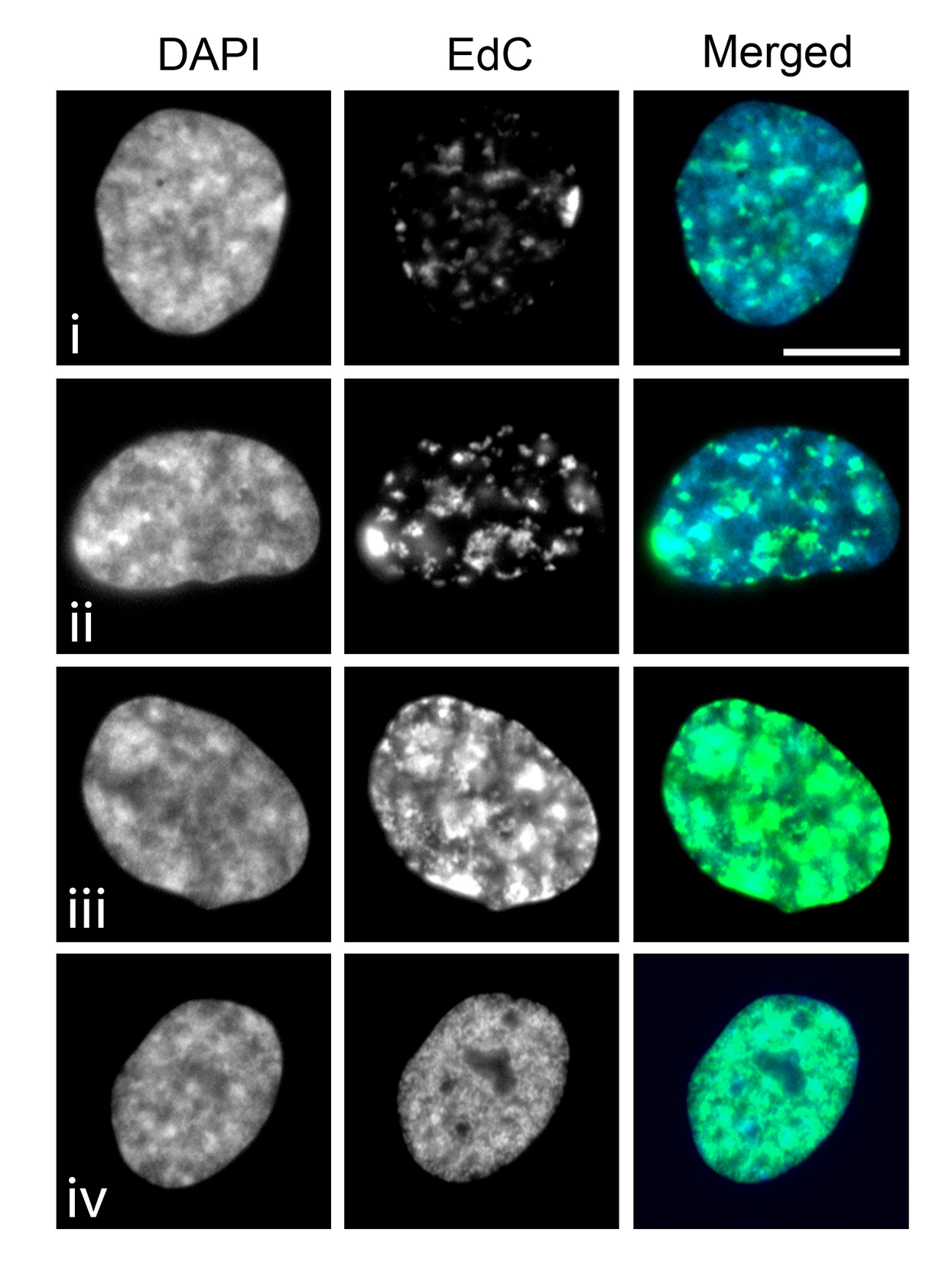

Supplement: S2 Fig — RPE-1 cells were pulsed for 4 h with 5 μM EdC, and processed. Incorporated EdC was detected by cycloaddition using Alexa 488-azide capture reagent and cells counterstained with DAPI. Examples of different localisation patterns in uninfected S-phase cells (labelled i-iv as discussed in the text) are illustrated (scale bar 10 μm). (TIF) [file ppat.1006721.s002.tif]

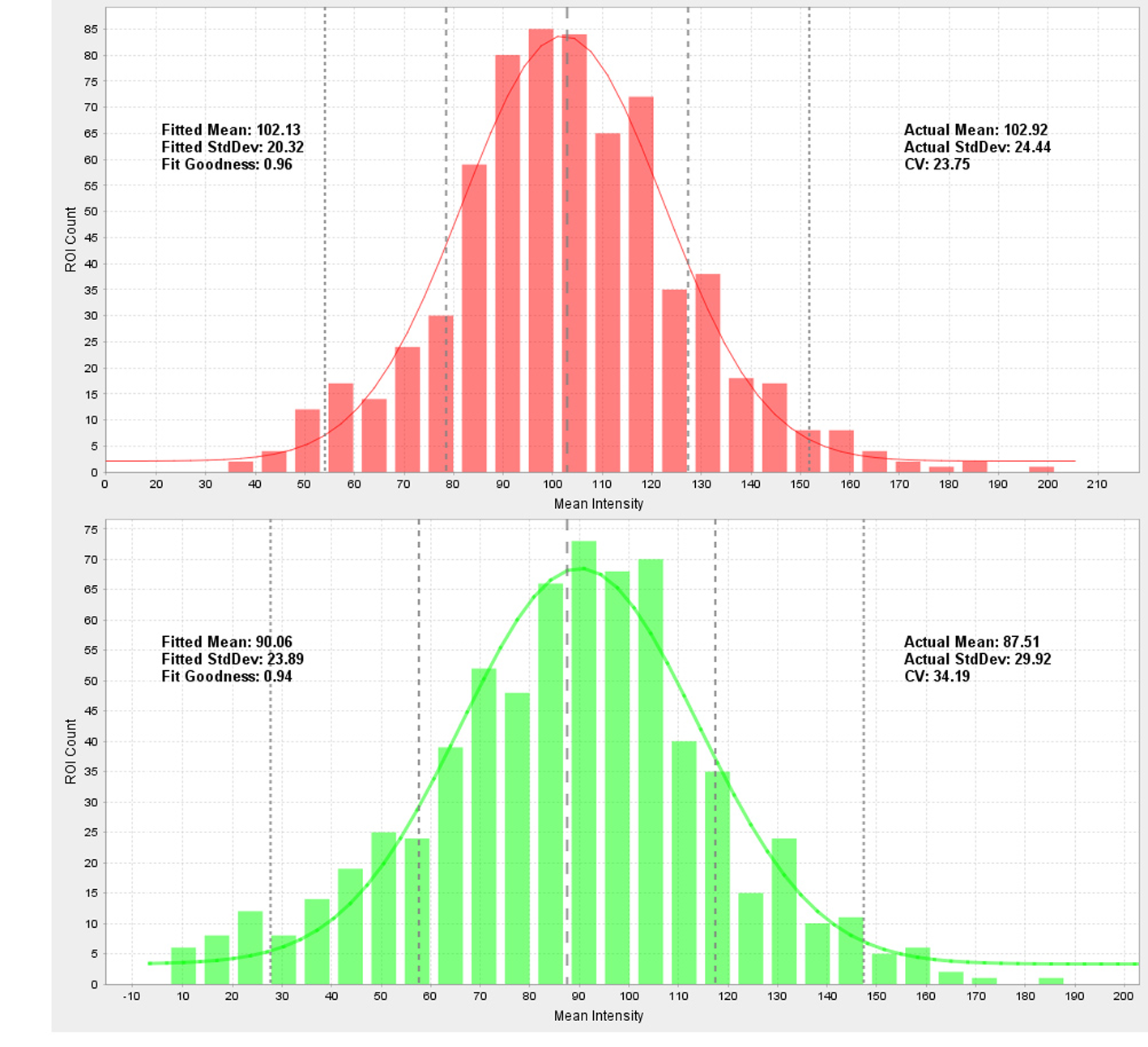

Supplement: S3 Fig — Quantitative analysis of individual particles was performed as described for Fig 4 and in materials and methods. We used a customised Image J plugin based on the find maxima protocol accompanied by pixel quantification in defined ROIs for each channel (top panel VP5, red; bottom panel EdC, green). Particles were scored positive by being not only above ROI normalised background threshold, but being at least 1 SD above that value. Frequency distributions of signal intensities for each channel of individual VP5 positive particles were quantitated with bin width automatically selected using the Friedman-Diaconis criteria for interquartile-ranges [74, 75]. The same bin width was used for both channels aligning means for both channels for ease of comparison of distributions. The raw mean and SD together with the coefficient of variance are reported on the right-hand side, with fitted means and SDs reported on the left hand side after Gaussian distributions were fitted to each channel frequency data using Image J curve fitter. (TIF) [file ppat.1006721.s003.tif]

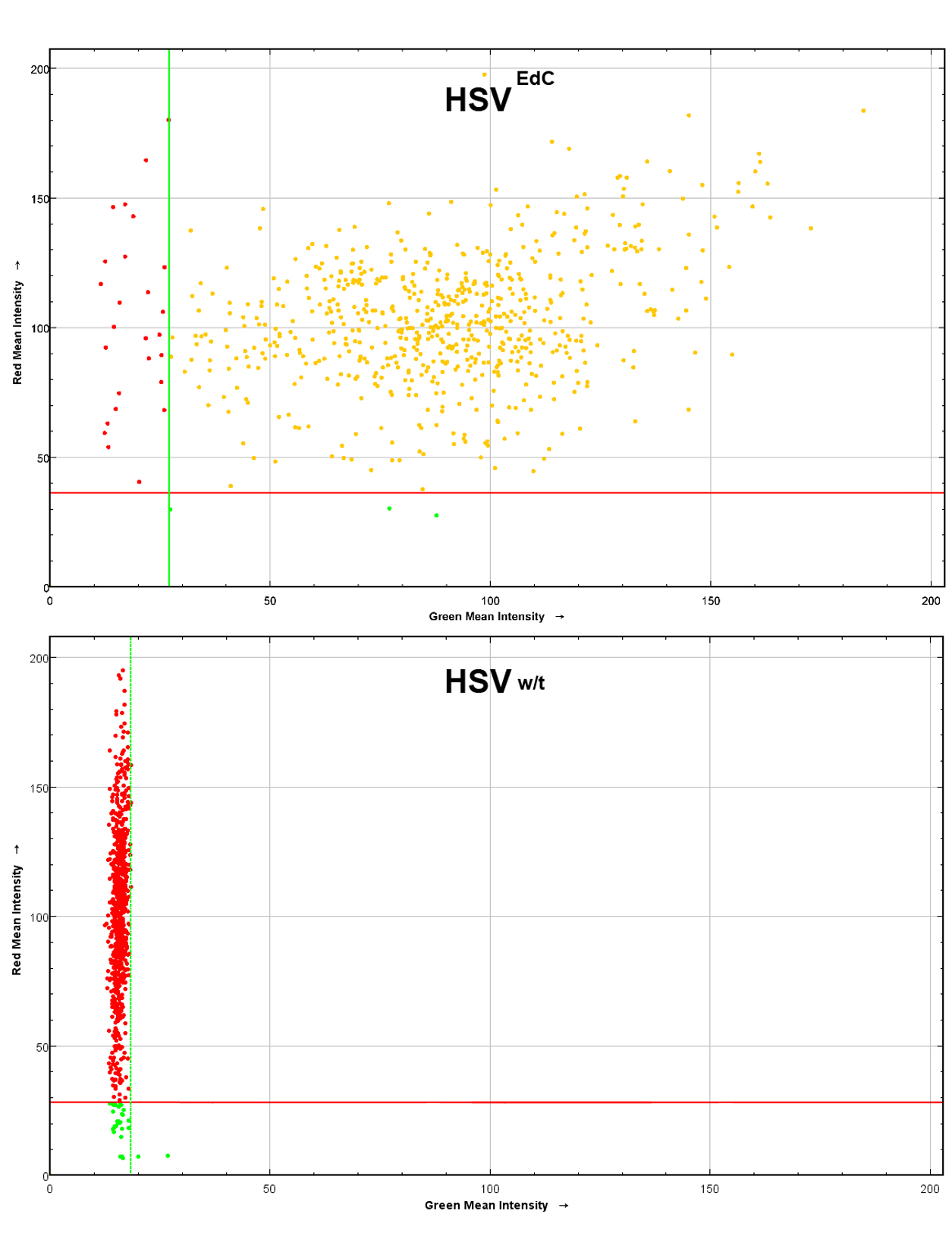

Supplement: S4 Fig — The same analysis used for overall frequency distributions of VP5 and DNA of the total particles (S3 Fig) was used to generate scatter plots where each dot represents an individual particle and its score for VP5 (Y-axis) and EdC (x-axis). The threshold for scoring positive is indicated by the solid lines in red or green. Note that thresholds are calculated with regard to individual images and background for data in each field and may be marginally different. Particles for HSVEdC that are positive for both signals are in the upper right quadrant and coded yellow. Particles which are VP5 positive and below threshold in green are coded red. For HSV w/t essentially no VP5 positive particle exhibited any significant green signal. (TIF) [file ppat.1006721.s004.tif]

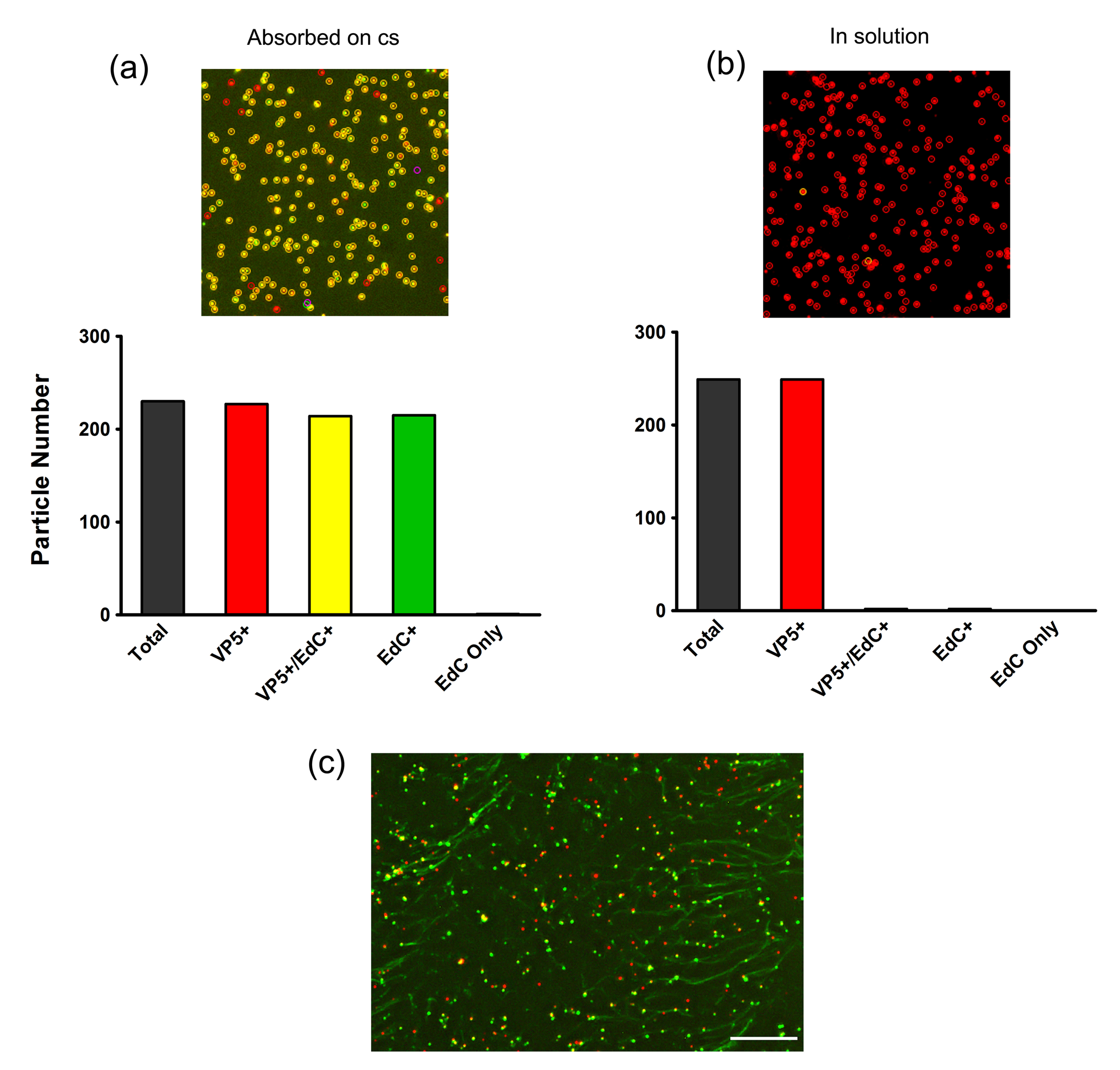

Supplement: S5 Fig — (a) HSV-1EdC virus particles were adsorbed onto borosilicate coverslips, detected by cycloaddition and immunofluorescence and analysed as described for Fig 4. (b) HSV-1EdC was examined by cycloaddition in solution prior to adsorption on coverslips. The reaction was then blocked by addition of 1 mM EDTA to chelate the copper catalyst and the sample then adsorbed to borosilicate coverslips, stained for VP5 and analysed by ImageJ. Graphs show quantified data of particles under each condition for VP5 and EdC. (c) HSV-1EdC was adsorbed to borosilicate coverslips as above and then heated to 70°C for 2 min before fixation and detection by cycloaddition and immunofluorescence for VP5. VP5, red; EdC, green (scale bar 10 μm). (TIF) [file ppat.1006721.s005.tif]

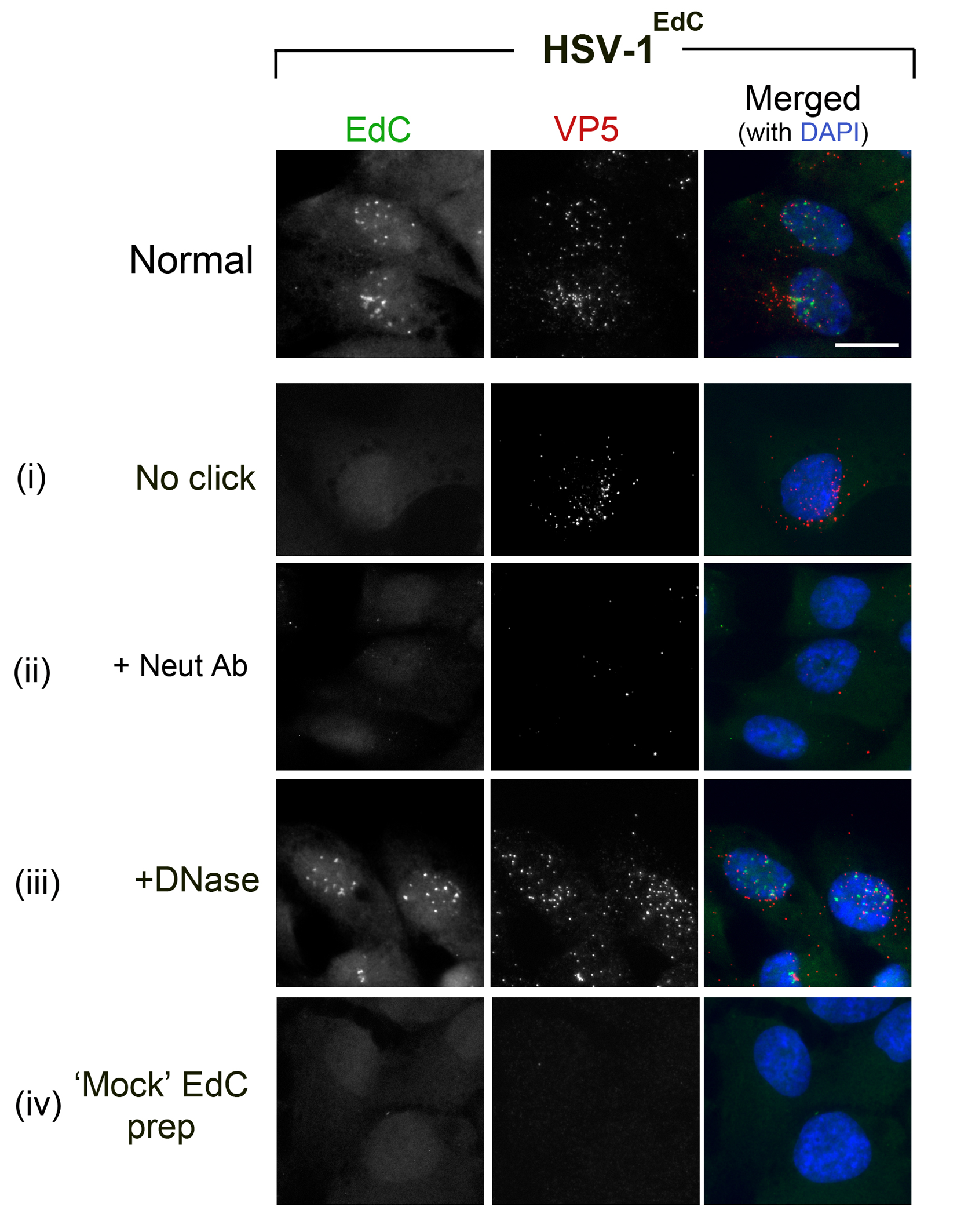

Supplement: S6 Fig — RPE-1 cells were infected with HSV-1EdC and fixed at 2 hpi for detection by cycloaddition and immunofluorescence for VP5 (scale bar 10 μm). Experimental variations from the normal process were as follows: (i) During the cycloaddition, Cu(I) was omitted from the reaction mixture; (ii) the virus inoculum was treated with clinical grade neutralizing antibody IVIg (100 mg/ml) for 0.5 hr at room temperature prior to infection; (iii) the inoculum was treated with DNase I (500 U/ml) for 0.5 h at 10°C prior to infection; (iv) cells were infected with a ‘mock’ inoculum which consisted of concentrated supernatant from uninfected RPE-1 cells pulsed and prepared as for an infected HSV-1EdC stock. (TIF) [file ppat.1006721.s006.tif]

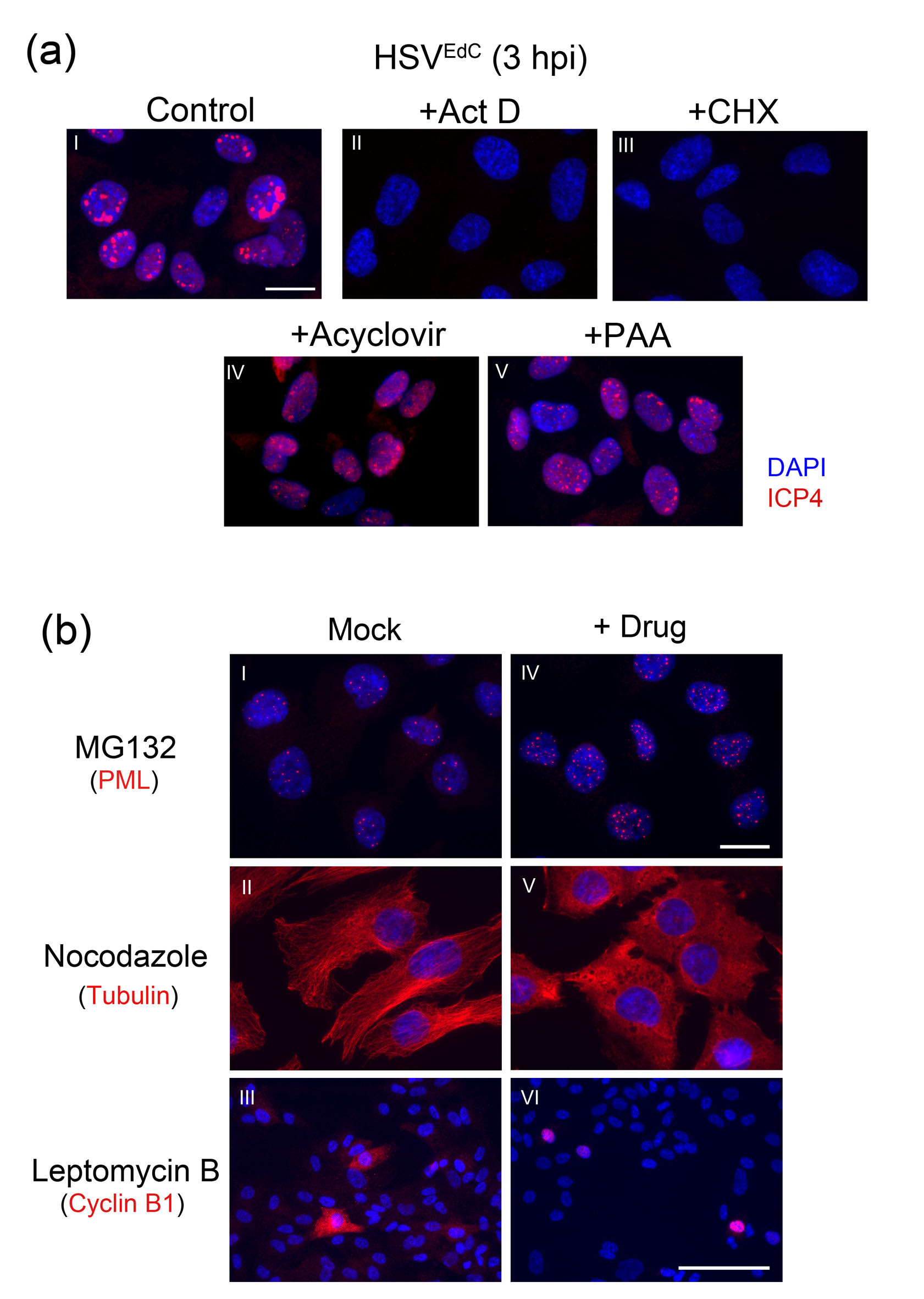

Supplement: S8 Fig — (a) Representative images of the localisation of ICP4 in cells infected with HSV-1EdC (moi 10) and either untreated or incubated in the presence of ActD (5 μg/ml), CHX (100 μg/ml), or ACV (500 μM), PAA (400 μg/ml), as indicated. Cells were fixed (3 hr post infection) and processed for immunofluorescence of ICP4. Scale bar 10 μm. (b) Uninfected cells were treated with MG132 (10 μM), nocodazole (2 μM), or Leptomycin B (20 nM) for 1.5 hr, as during analysis of infection, then fixed and processed for immunofluorescence for PML, α-tubulin, or cyclin B1 respectively (red channel). Cells were counterstained with DAPI. Panels I and IV show MG132 treatment increases PML number and size per nuclei; panels II and V demonstrate microtubule depolymerisation upon nocodazole treatment; panels III and VI show inhibition of nuclear export and nuclear accumulation of cyclin B1. Scale bar for MG132 and nocodazole panels, 10 μm; scale bar for Leptomycin treatment, 100 μm (TIF) [file ppat.1006721.s008.tif]

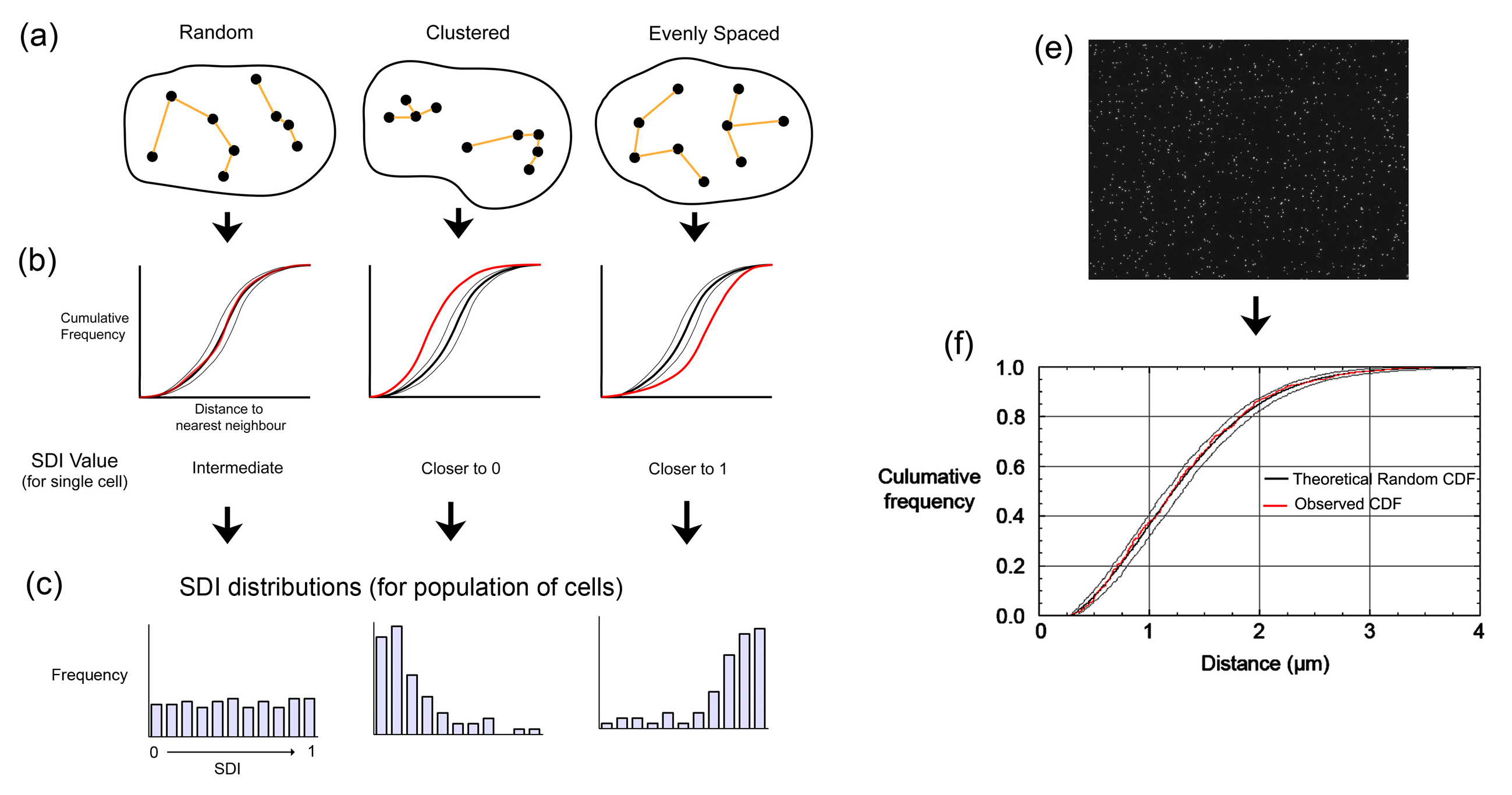

Supplement: S9 Fig — (a) Schematic illustration of theoretical random, clustered or evenly spaced patterns of foci in an individual cell. The spatial distribution analysis examines the overall distribution of inter-point distances, including any local clusters, and calculates whether there is any evidence for a non-random distribution in the population of cells. (b) The distances between every point and its nearest neighbour are generated as a cumulative distribution function (CDF) of those distances (see materials and methods). A theoretical CDF for a truly random distribution (black line; 95% confidence limits, grey lines) is generated (specific for each nucleus and number of foci) and then compared with the actual distribution obtained for the test set (red line). For an actual random pattern the red line will be close to the black, while for clustered and evenly spaced the red line will deviate left or right respectively. This difference is transformed to a spatial distribution index (SDI). Point patterns that tend to be clustered have a SDI closer to 0 while evenly spaced patterns have a SDI closer to 1. (c) The corresponding SDI frequency distributions between 0 and 1 for populations of cells. The CDFs of SDIs of different populations are compared using the Kolmogorov-Smirnoff (KS) test, which is non-parametric and distribution free. A p-value for the difference between the two populations is calculated, as well as the D statistic which is the largest deviation between the two CDFs. (e) Capsids applied on coverslips were fixed, stained with anti-VP5 and images processed to calculate the SDI, expected to be random. (f) The results compare the theoretical cumulative inter-point distances for a random distribution (black line; 95% confidence limits, grey lines) with the actual distribution obtained for the capsids (red line). The capsid distribution precisely conforms to a random distribution. Note while clusters can be observed, such clusters will occur randomly, by definition. The [file ppat.1006721.s009.tif]
